# Fundamental Limits of Forced Asynchronous Spiking with Integrate and Fire Dynamics

**DOI:** 10.1186/s13408-017-0053-5

**Published:** 2017-10-11

**Authors:** Anirban Nandi, Heinz Schättler, Jason T. Ritt, ShiNung Ching

**Affiliations:** 1 Electrical and Systems Engineering, Washington University in St. Louis, St. Louis, MO USA; 2 Department of Biomedical Engineering, Boston University, Boston, MA USA; 3 Department of Biomedical Engineering, Washington University in St. Louis, St. Louis, MO USA

**Keywords:** Time optimal control, Spike pattern, Selective spiking

## Introduction

The manipulation of networks of neurons in the brain through the use of extrinsic controls—neurocontrol—is a key problem in experimental neuroscience [1]. Such capability has the potential to enable new and important study of questions in neural coding or how the firing activity of brain cells determines their ability to carry and process information [2]. Moreover, improving the use of neurostimulation may aid the refinement of how such technology is used in clinical settings [3, 4].

The use of stimulation in the study of neural coding is itself an established paradigm in neuroscience. The general idea is straightforward: by inducing neural activity and observing the consequent behavior of the organism, we can infer the functional role of the region in question. For example, cortical microstimulation of certain brain regions has been shown to induce behavioral changes in the context of perceptual tasks such as visual decision-making [5, 6]. Recently, several key advances in neurostimulation technology, such as the advent of optogenetics [7], have made neurocontrol possible at unprecedented spatial scales. Thus, experimentalists are able to assess the functional role not simply of different neural populations, but potentially of specific neurons and the timing of their spikes. That is, it may be possible to test the long-standing neural coding hypothesis that spike timing is crucial to information processing [8].

**Fig. 1:**
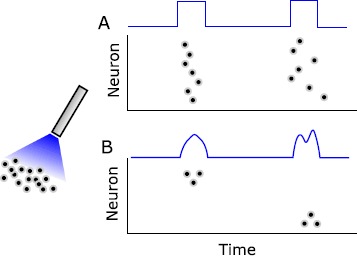
Underactuated neurocontrol schema. Most neurostimulation modalities are underactuated, wherein a single stimulation source impinges on orders-of-magnitude greater numbers of neurons. **(A)** The use of such stimulation has historically been limited to perturbative paradigms, wherein pulse-type inputs are used to create bulk population responses without fine temporal structure. **(B)** Increasingly, experimentalists seek to induce more precise spiking patterns in specific subsets of the population, which may necessitate the design of nuanced stimulation waveforms.

Currently, however, these hardware instantiations are typically used in perturbative paradigms wherein “pulses” of input are used to alter neural firing in a bulk manner (see Fig. 1) that does not control the precise timing of individual neuronal spikes. Formal control analysis or design in this context, though desired, is not well studied [9]. Thus, there is a need for formal mathematical analysis regarding the fundamental limits of such stimulation, particularly as it pertains to the feasibility of inducing precisely timed spiking activity in neural populations (Fig. 1).

### Prior Work in Neuronal Control

The control of neural activity has received substantial attention in the context of oscillations and synchronization, spurred in large part by interest in clinical brain stimulation for motor disorders [10, 11]. The objective in this class of neurocontrol problem is generally the forced splaying of neural phases (i.e., desynchronization), wherein neurons are typically modeled using phase oscillator formalisms (e.g., [12–18]). Alternatively, others have approached the problem of desynchronization from the perspective of physiological and instrumentation constraints, favoring methods involving strictly pulsatile stimulation [19–22].

In contrast, we consider herein the mathematical problem of asynchronous neurocontrol (i.e., control neural spiking without overt rhythmicity), iIn other words, forcing a neuron to spike but not necessarily periodically. The other key distinction of our work is that we consider a neuronal-level objective (i.e., spiking and spike timing) versus a population-level objective (i.e., synchronization or desynchronization). We have previously provided early formulations of this problem and highlighted key analytical challenges in the development of controllability analysis for spiking models [23, 24]. Other works regarding formal control design include optimal control design for a single neuron [25] and using statistical modeling frameworks [26, 27].

### Neurocontrol with Common Input

A key challenge associated with neurocontrol is *underactuation*, wherein a small number of inputs (in many current implementations, a single input) impinges on an orders-of-magnitude greater number of neurons [23], as schematized in Fig. 1. In other words, individual neurons are not addressed via independent inputs, but rather a common one. This challenge is ubiquitous across stimulation modalities and is, perhaps, the major constraint that has restricted the use of neurostimulation to the aforementioned perturbative paradigms. In the context of the discussed oscillatory objectives, some progress has been made on solving control problems such as entrainment and synchronization in the presence of underactuation [28–31]. However, this issue is unresolved in the case of asynchronous timed spike control objectives, such as those we consider herein. Current and foreseeable neurostimulation technologies are likely to face the challenge of underactuation, especially for *in vivo* instantiations.

### Specific Contributions

In this paper, we address the problem of time-optimal control of spiking in pairs of Leaky Integrate-and-Fire (LIF) neurons, where the desired spiking is *selective*, that is, certain neurons spike while others remain silent. We specifically focus on the case where two neurons receive a common input, which, as mentioned before, is a key constraint in the practical design of neurocontrol methods. Our major contributions are in the characterization of fundamental limitations for neuron-level control as revealed through a formal mathematical analysis. This treatment leads to the postulation of practical neurocontrol design strategies. Specifically, we provide:

1. The formal synthesis of time-optimal selective spiking solutions in pairs of LIF neurons. The synthesis involves application of the Pontryagin maximum principle, but with several nontrivial caveats due to the selectivity specification, which leads to state constraints. We prove that the optimal solution in this case involves use of the so-called boundary control, associated with the state constraints. Sufficient conditions for optimality are verified.
2. The formal synthesis for time-optimal control of longer sequences of spikes. Here, the solution is derived via dynamic programming, but again with several nontrivial developments due to nondifferentiability of the value function. In particular, we prove the nonexistence of an optimal solution for specific classes of spike sequences.
3. The development of design methods for timed patterns of spikes. In this case, there is no unique optimal solution. Nevertheless, we derive a greedy algorithm that can provide near-perfect construction of patterns under specified conditions. Finally, we evaluate the performance of our control design when the system is subjected to noise and disturbances.

Our presentation and discussion on fundamental optimal control analysis and design work toward the overall goal of understanding the limits of neurocontrol. We illustrate several interesting control phenomena that arise due to the peculiarity of spiking dynamics. Specifically, the problem considered, although ostensibly simple, leads to several interesting features in the optimal control synthesis due to state constraints.

## Background and Methods

### Definitions: Spike Sequence and Pattern Control

We begin by formally defining the notions of spike sequences and patterns, which will facilitate our approach to spike timing control.

#### Definition 1

Spike Sequence

In a population of *N* neurons, an M-spike sequence is a vector

1 $$ \varSigma_{S} = [ \sigma_{1}, \sigma_{2},\dots, \sigma_{M} ] , $$

where $\sigma_{k} \in \{ 1,2,\dots,N \} $ indicates the neuron that produces the *k*th spike in the sequence.

#### Definition 2

Spike Pattern

In a population of *N* neurons, an M-spike pattern is a sequence with timing, that is,

2 $$ \varSigma_{P} = \bigl[ (\sigma_{1},t_{1}), (\sigma_{2},t_{2}),\dots,( \sigma_{M},t_{M}) \bigr] , $$

where $\sigma_{k} \in \{ 1,2,\ldots,N \} $ indicates the neuron which produces the *k*th spike at time $t_{k}>0$, where $t_{1}< t_{2}< \cdots<t_{M}$.

The goal of this paper is to provide a set of fundamental characterizations regarding the time-optimal control of spike sequences and patterns.

### Model Formulation

We proceed with the model formulation, starting with the base model and then adding synaptic coupling between neurons.

#### Base Model

**Fig. 2:**
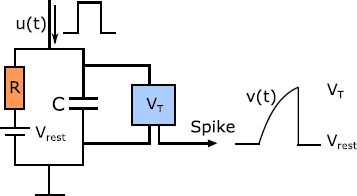
The leaky integrate-and-fire circuit. The membrane potential rises under the stimulus $u(t)$ until it hits the threshold $V_{T}$, at this point $v(t)$ is artificially reset to $V_{\mathrm{rest}}$ and a spike is said to be generated. We also show a cartoon of the possible voltage trace of the neuron under a rectangular pulse input.

The integrate-and-fire neuron is a well-established model in computational neuroscience [32, 33]. The circuit of this model is shown in Fig. 2, where a capacitor *C* and resistance *R* (modeling the capacitive and resistive properties of the cell membrane) are in parallel, with $u(t)$ being the external stimulus. Denoting the membrane potential as $v(t)$, the charge deposited on the capacitor is $q = Cv$, and therefore the current is given by $I_{C} = C \frac{\mathrm{d}v}{\mathrm{d}t}$, leading to the linear dynamics

3 $$ C \frac{{\mathrm{d}v(t)}}{{\mathrm{d}t}} = \frac{{V_{\mathrm{rest}}}-v(t)}{R} + \beta u(t) + I_{\mathrm{syn}}, $$

where $V_{\mathrm{rest}}$ is the resting potential, and $\kappa _{\mathrm{m}} = RC$ is the membrane time constant. Here, $I_{\mathrm{syn}}$ denotes synaptic input entering from other neurons. We also introduce a parameter *β* that encapsulates the effectiveness of the external input $u(t)$ for each neuron.

*Spike generation.* In this model, a spike is said to be generated at time $t_{s}$ if the membrane potential reaches a predetermined threshold voltage $V_{T}$. Upon emitting a spike, the membrane potential is reset to $V_{\mathrm{rest}}$. Thus, spike generation is governed by the discontinuous resetting rule

4 $$ v\bigl(t_{s}^{-}\bigr) = V_{T} \quad \to\quad v\bigl(t_{s}^{+}\bigr)=V_{\mathrm{rest}}. $$

*Model normalization.* In what follows, we assume that $V_{\mathrm{rest}} = 0$. This normalizing assumption is not restrictive, since it can be readily achieved by a simple translation in the coordinate system, that is, $v \leftarrow(v - V_{\mathrm{rest}})$, ${V}_{T} \leftarrow(V_{T} - V_{\mathrm{rest}})$.

#### Synaptic Coupling

We build an approximate model of synaptic coupling based on the standard formulations in [33]. Key to this formulation is the notion of impulsive coupling, wherein the major effect of $I_{\mathrm{syn}}$ occurs during a brief time window following an afferent spike (i.e., a spike from another neuron). Following a reduction of continuous synaptic models (see Appendix A.1), we formulate $I_{\mathrm{syn}}$ as

5 $$ I_{\mathrm{syn}}(t) = \rho_{\mathrm{syn}}(t) \sum _{t_{s} \in \mathcal{T}}\delta (t-t_{s}), $$

where $\mathcal{T}$ denotes the set of all afferent spike times, and $\rho_{\mathrm{syn}}(t)$ is a synaptic constant that depends on the specific parameters of the neuron. If all neurons remain below the threshold, then $I_{\mathrm{syn}} \equiv0$.

Thus, the effect of a synaptic event on the postsynaptic neuron can be understood as an instantaneous rise in voltage that occurs only when a neighboring, connected neuron fires a spike. Knowing this rise can allow us to insulate neurons from each other in the spike control problem, formulated in the next section.

### Problem Formulation: Minimum Time Selective Spiking

In this paper, we study three base problems pertaining to the design of $u(t)$ to create structured spiking patterns in populations of two LIF neurons of the form (3). We first consider the problem of time-optimal sequence control, that is, inducing target sequences with minimal temporal spacing between the beginning and end of the sequence. It turns out that this problem amounts to an analysis of *selective spiking*. We formulate a canonical version of this problem in two dimensions.

#### Problem 1

P1: Pairwise time-optimal selective spiking with synaptic guard

Consider two coupled LIF neurons of the form (3):

6 $$\begin{aligned} \begin{bmatrix} \dot{v}_{1}\\ \dot{v}_{2} \end{bmatrix} &= \begin{bmatrix} -a_{1} &0\\ 0 &-a_{2} \end{bmatrix} \begin{bmatrix} {v}_{1}\\ {v}_{2} \end{bmatrix} + \begin{bmatrix} b_{1}\\ b_{2} \end{bmatrix} u + \begin{bmatrix} I_{\mathrm{syn}_{1}}\\ I_{\mathrm{syn}_{2}} \end{bmatrix} \\ &\equiv f(\mathbf{v}, u, I_{\mathrm{syn}}) = A\mathbf{v}+bu+\textbf{I} _{\mathrm{syn}}, \end{aligned}$$

where $\mathbf{v} = [v_{1}\ v_{2}]^{T}$, $a_{i} = \frac{1}{R_{i}C _{i}}$, $b_{i}= \frac{\beta_{i}}{C_{i}}$, $a_{i}, b_{i} >0$, and $I_{\mathrm{syn}_{i}}$ are impulsive synaptic inputs of the form (5) for $i = 1, 2$. Find the control input $u(t)$ such that

7 $$ v_{1} ( \tau ) = {V_{T}},\qquad v_{2} ( t ) \le{V_{G}} < {V_{T}}, \quad \forall t \in [ 0,\tau ] , $$

with arbitrary initial condition $\mathbf{v}(0) \in\mathcal{G}$, where

8 $$ \mathcal{G}=\bigl\{ (v_{1},v_{2}):0\leq v_{1} \leq V_{T},0\leq v_{2} \leq V _{G}\bigr\} , $$

and $u(t)$ solves the time-optimization

9 $$ \text{minimize}\quad \mathbb{J}(u) =\int_{0}^{\tau}\mathrm{d}t $$

over all measurable functions *u* that take values in the control set, where $\mathcal{U}$ is this set of admissible inputs.

Taken together, (7)–(9) imply that Neuron 1 produces a spike before Neuron 2 and that, under (7), the spike occurs in minimum time.

*Functional decoupling of the network via guard* $V_{G}$ *.* The parameter $V_{G}$ in (7), referred to as a synaptic guard, is key to selectivity. It ensures that Neuron 2 remains below threshold and, further, is insulated from the synaptic effect due to the induced spike in Neuron 1, that is,

10 $$ \begin{aligned} &V_{G} < V_{T} - \bar{\rho}_{\mathrm{syn}}; \quad \bar{\rho}_{\mathrm {syn}} = \sup_{t} \rho_{\mathrm{syn}}(t), \end{aligned} $$

where $\rho_{\mathrm{syn}}(t)$ is the synaptic contribution to the post-synaptic neuron (here, Neuron 2) and is derived in Appendix A.1. The guard, in essence, keeps the nonselected neuron sufficiently away from its own threshold so as not to produce an undesired, collateral spike.

It is important to note that in solving (*P1*), it is sufficient to consider the dynamics in (6) as

11 $$ \dot{\mathbf{v}} = f(\mathbf{v}, u, 0) \equiv f(\mathbf{v}, u) = A \mathbf{v}+bu, $$

since both neurons are below threshold for the duration of the synthesis. Despite this simplification in the dynamics, the selectivity/guard criterion (7) poses a key challenge, that is, it is not sufficient to simply fire Neuron 1 in minimum time, since doing so may in general cause Neuron 2 to fire an undesired spike. Mathematically, (7) functions as a state constraint that, as we will see, leads to several complications in the optimal synthesis.

If the problem has a solution for either choice of neuron labeling, then the population is said to be *pairwise feasible*, that is, either neuron can be made to spike selectively.

#### Problem 2

P2: Pairwise time-optimal selective sequencing

For the two-neuron network in (11), find the control input that achieves any *M*-spike target spike sequence $\varSigma_{S}$ time optimally, that is,

12 $$ \mathop{\text{minimize}}_{u \in\mathcal{U}}\quad \mathbb{J}(u) =\int_{0}^{\tau_{1}}\mathrm{d}t + \cdots+ \int_{\tau_{M-1}}^{\tau_{M}} \mathrm{d}t $$

such that

13 $$ \begin{aligned} v_{\sigma_{k}}(\tau_{k})&= V_{T}, \\ v_{\hat{\sigma}_{k}}(t) &\leq V _{G}, \quad \forall t\in[\tau_{k-1}, \tau_{k}],\mathbf{v}(0) \in\mathcal{G}, \hat{\sigma}_{k} = \varOmega \backslash\sigma_{k}, \text{where }\varOmega= \{1,2\}, \\ k& = 1,\ldots, M, \text{and } \tau _{0} = 0. \end{aligned} $$

The key complication here is the nondifferentiability of the value function within the dynamic programming, as well as the spike discontinuity (4).

#### Problem 3

P3: Pairwise time-optimal selective patterning

Considering the same model in (11), find the control that induces the spiking in the two neurons according to the times specified in the target pattern $\varSigma_{P}$, constrained by the underlying sequence. Mathematically,

14 $$ \begin{aligned} & \mathop{\text{minimize}}_{u \in\mathcal{U}} & & \mathbb{J}(u) = \sum_{k=1}^{M} \biggl( (t_{k}- t_{k-1})- \int_{\tau_{k-1}}^{\tau_{k}} \mathrm{d}t \biggr) ^{2} \end{aligned} $$

with the same constraints as described in (13) and $t_{0} = \tau_{0}= 0$. Note that $t_{k}$ are the desired spike times, and $\tau_{k}$ are the actual spike times.

## Minimum Time Selective Spiking

We consider the minimum-time selective spiking problem *P1*. We assume, without loss of generality, that the neurons are labeled so that the objective is to fire Neuron 1. It turns out that the solution to this problem depends on the ratio (see Appendix A.2)

15 $$ \gamma_{1} = \frac{b_{1}a_{2}}{b_{2}a_{1}}, $$

which we treat in two separate cases corresponding to $\gamma_{1} \lessgtr\frac{V_{T}}{V_{G}}$.

As we will show in the following sections, for $\gamma_{1} > \frac{V _{T}}{V_{G}}$, that is, Case 1, selective spiking can always be accomplished. However, if $\gamma_{1} \leq\frac{V_{T}}{V_{G}}$, that is, Case 2, a solution may not exist, and pairwise feasibility is not guaranteed.

### Selective Spiking, Case 1: $\gamma_{1} > \frac{V_{T}}{V _{G}}$

#### Proposition 1

*Consider the two*-*neuron network* (11), *where*

16 $$ \gamma_{1} > \frac{V_{T}}{V_{G}}. $$

*Assume that the set of admissible controls* $\mathcal{U}$ *forms a box constraint of the form* $\mathcal{U} = [0,U]$, *and we take as given the initial conditions* $v_{i}(0)< V_{G}$, $i = 1,2$. *The time optimal feedback control* $u^{*} \in\mathcal{U}$ *for the selective spiking problem P*1 *for Neuron *1 *is given by*

17 $$ u^{\ast} = \textstyle\begin{cases} U& \textit{for }v_{2}< V_{G}, \\ u_{\mathrm{arc}}& \textit{for } v_{2}=V_{G}, \end{cases} $$

*where* $u_{\mathrm{arc}}=\frac{a_{2}}{b_{2}} V_{G}$ *is the unique control that keeps* $v_{2}(t)=V_{G}$ *invariant*. *Moreover*, *such a control always exists*. *Thus*, *optimal controls are either given by a constant control at maximum value*, $u^{*}(t) \equiv U$, *if the state space constraint does not become active*, *or if the corresponding trajectory meets the state space constraint*, *then optimal controls are a concatenation of a segment for the maximum control until the state constraint is reached followed by a constant boundary control* $u^{*}(t) = u_{\mathrm{arc}}$ *until the terminal value* $v_{1}=V_{T}$ *is reached*.

#### Proof

Necessary conditions for optimality for problem *P1* are given by the Pontryagin maximum principle. In the presence of state space constraints, these take a rather complicated form (the multipliers associated with the state space constraint are measures). The problem considered here, however, is simpler, and instead of analyzing those conditions, we shall define a synthesis of extremal controlled trajectories through a direct construction and then verify the optimality of the synthesis. In particular, there is no need to consider possible degeneracies that in principle are allowed by necessary conditions for optimality (e.g., abnormal extremals, etc.).

*Synthesis Construction.* We want to solve the optimal control problem *P1* on the set $\mathcal{G}$ in (8). We first treat the problem in the absence of the state constraint and define the Hamiltonian function as

18 $$ \mathcal{H}(\lambda,\textbf{v}, u) = 1 + \lambda\cdot f( \textbf{v},u) = 1+ \lambda\cdot(A\textbf{v} +bu). $$

According to the maximum principle, as long as no state space constraints are active, the multiplier *λ* is a solution to the adjoint equation

19 $$ \dot{\lambda}(t) = -\lambda(t) A, $$

and the optimal control minimizes the Hamiltonian over the control set $[0,U]$. The solutions of (19) are of the form

20 $$ \lambda_{1}(t) = c_{1}\mathrm{e}^{a_{1}t},\qquad \lambda_{2}(t) = c_{2}\mathrm{e}^{a_{2}t} $$

for some constants $c_{1}$ and $c_{2}$, and thus

21 $$ u^{\ast}_{\mathrm{NoGuard}}(t) = \textstyle\begin{cases} U &\text{if $\varPhi(t) < 0$,} \\ 0 & \text{if $\varPhi(t) >0$,} \end{cases} $$

with

22 $$ \varPhi(t) = b_{1}\lambda_{1}(t) + b_{2}\lambda_{2}(t) $$

as the switching function. The terminal constraint is defined by $\psi(\tau,\textbf{v}) = v_{1}(\tau)-V_{T}$, and the transversality condition [34, Sect. 2.2] of the maximum principle implies that $\lambda(\tau)=[\nu \;\; 0]$ where *ν* is some multiplier. This gives us $c_{2} = 0$, and thus the switching function has a constant sign in the absence of the guard constraint. Hence the optimal control is simply a BANG, that is, the maximal input.

With the state constraint (the guard), there can be switching in the optimal control, and we need to consider two subcases: trajectories that do or do not hit the boundary $v_{2}=V_{G}$. For *A* with real eigenvalues, the optimal controls of linear single input control systems are BANG-BANG with at most $n-1$ switchings (where *n* is the dimension of the system; here $n = 2$) [34], and we must have $u>0$ at the spike time (otherwise, **v** would be decaying). We thus consider controls only of the form

23 $$ u = \textstyle\begin{cases} 0 &\text{for $t \leq\hat{t}$ where $v_{1}(\hat{t}) < V_{T}$}, \\ U &\text{for $\hat{t} < t \leq\tau$}. \end{cases} $$

These define a smooth flow of extremal controlled trajectories as long as the state space constraint is not violated. If the extremals hit the state constraint boundary, then the control must switch to the boundary control $u_{\mathrm{arc}}$ that keeps the system from exceeding the constraint:

24 $$ u_{\mathrm{arc}}= \frac{a_{2}V_{G}}{b_{2}}. $$

However, we need to verify whether this boundary control $u_{\mathrm{arc}}$ will eventually bring Neuron 1 to threshold. For $v_{1} = V_{T}$ and $u = u_{\mathrm{arc}}$, we have

25 $$ \dot{v}_{1}= -a_{1}V_{T} + b_{1}\frac{a_{2}V_{G}}{b_{2}}>0, $$

where the inequality holds by our assumption on $\gamma_{1}$. Now, if (25) holds, then in fact $\dot{v}_{1} > 0$ for all $v_{1} \in[0, V_{T}]$ under the boundary control, and $v_{1}$ will eventually reach threshold.

Thus for appropriate initial conditions, applying the maximal input $u(t)=U$ produces a spike in Neuron 1 without hitting the Neuron 2 guard. For the remaining initial conditions, we construct a control that applies maximal input until the guard is reached and then drops to $u_{\mathrm{arc}}$ until $v_{1}$ hits threshold. Note that we do not need to employ the zero control in (23), so we may take $\hat{t}=0$ (the possibility of additional switching will arise in the next section under the alternative case for $\gamma_{1}$). Thus the control (17) will produce a spike in Neuron 1 without inducing a spike in Neuron 2 across all initial conditions. *This concludes the synthesis construction.*

*Proof of Optimality.* The optimality of this control follows from regular synthesis-type sufficient conditions for optimality, and we briefly outline the reasoning. The value or cost-to-go function of this synthesis is continuous but not differentiable on the curve that separates initial states for which the trajectory includes a boundary segment from those that do not. The curve *Γ* that separates these two regions is defined by the set of initial conditions that hit the final condition $\textbf{v}(\tau)=[V_{T}\ V_{G}]^{T}$ under the BANG control $u(t)=U$. To find this curve, we first explicitly compute the time for $v_{1}$ to hit threshold,

26 $$ \tau= \frac{1}{a_{1}}\log \biggl( \frac{\frac{b_{1}}{a_{1}}U-v_{1}(0)}{ \frac{b_{1}}{a_{1}}U-V_{T}} \biggr) \equiv\frac{1}{a_{1}}\log \bigl( E\bigl(v _{1}(0) \bigr)^{-1} \bigr) , $$

where for convenience we define

27 $$ E(v) = \frac{\frac{b_{1}}{a_{1}}U-V_{T}}{\frac{b_{1}}{a_{1}}U-v}. $$

We then eliminate *τ* by solving explicitly for $v_{2}(t)$ with the final condition $v_{2}(\tau) = V_{G}$

28 $$ V_{G} = E\bigl(v_{1}(0) \bigr)^{\frac{a_{2}}{a_{1}}}v_{2}(0) + \frac{b_{2}}{a _{2}}U \bigl( 1 - E \bigl(v_{1}(0)\bigr)^{\frac{a_{2}}{a_{1}}} \bigr) , $$

to find the separatrix as

29 $$ \begin{aligned} \varGamma= \biggl\{ \textbf{v} \in \mathcal{G} : E(v_{1})^{\frac{a_{2}}{a _{1}}}v_{2} + \frac{b_{2}}{a_{2}}U \bigl( 1 - E(v_{1})^{\frac{a_{2}}{a _{1}}} \bigr) - V_{G} =0 \biggr\} . \end{aligned} $$

We define the region $\varGamma_{-}$ as bounded between *Γ* and $v_{1}=V_{T}$ inclusive, and the region $\varGamma_{+}=\mathcal {G}\setminus \varGamma_{-}$. Thus, $\varGamma_{+}$ includes all initial conditions whose trajectories include a boundary arc, whereas initial conditions in $\varGamma_{-}$ can be driven to threshold directly at maximum input.

The value function corresponding to this synthesis is

30 $$ \mathcal{V} = \textstyle\begin{cases} \mathcal{V}_{-}(\textbf{v}) & \text{for $\textbf{v} \in\varGamma_{-}$}, \\ \mathcal{V}_{+}(\textbf{v}) & \text{for $\textbf{v} \in\varGamma_{+}$}. \end{cases} $$

For trajectories without a boundary arc, the value is just the spike time under maximal input, calculated as in (26),

31 $$ \mathcal{V}_{-}(\textbf{v}) = \frac{1}{a_{1}}\log \bigl(E(v_{1})^{-1}\bigr). $$

The calculation of the value $\mathcal{V}_{+}(\textbf{v})$ involves two steps: the time $t_{g}$ for Neuron 2 to reach the guard voltage, plus the time $t_{\mathrm{th}}$ for Neuron 1 to attain the threshold $V_{T}$ under the boundary arc control. By direct calculation,

32 $$\begin{aligned} \mathcal{V}_{+}(\textbf{v}) &= t_{g}+t_{\mathrm{th}} \\ &=\frac{1}{a_{2}} \log \biggl(\frac{\frac{b_{2}}{a_{2}}U-v_{2}}{\frac{b_{2}}{a_{2}}U-V _{G}} \biggr) + \frac{1}{a_{1}} \log \biggl(\frac{ \frac{b_{1}}{a_{1}}u_{\mathrm{arc}}-v_{1}(t_{g})}{\frac{b_{1}}{a_{1}}u_{\mathrm{arc}}-V _{T}} \biggr), \end{aligned}$$

where

33 $$ \begin{aligned} v_{1}(t_{g}) = \biggl(\frac{\frac{b_{2}}{a_{2}}U-V_{G}}{ \frac{b_{2}}{a_{2}}U-v_{2}} \biggr)^{\frac{a_{1}}{a_{2}}}v_{1} + \frac{b _{1}}{a_{1}}U \biggl(1 - \biggl(\frac{\frac{b_{2}}{a_{2}}U-V_{G}}{ \frac{b_{2}}{a_{2}}U-v_{2}} \biggr)^{\frac{a_{1}}{a_{2}}} \biggr) \end{aligned} $$

is the Neuron 1 voltage at the time $t_{g}$, that is, when the trajectory hits the Neuron 2 guard.

It is clear from the construction that $\mathcal{V}$ is continuously differentiable in the interior of $\mathcal{G}$ away from the curve *Γ*. We now show that on *Γ*, $\mathcal{V}$ remains continuous, but is no longer differentiable. Substituting $v_{2}$ from (29) into (33) yields

34 $$ v_{1}(t_{g}) = \frac{V_{T}-\frac{b_{1}}{a_{1}}U}{v_{1}- \frac{b_{1}}{a_{1}}U}v_{1} + \frac{b_{1}}{a_{1}}U \biggl(1 - \frac{V _{T}-\frac{b_{1}}{a_{1}}U}{v_{1}-\frac{b_{1}}{a_{1}}U} \biggr)= V _{T}. $$

Hence (32) reduces to

35 $$ \mathcal{V}_{+}(\textbf{v}) = t_{g}= \frac{1}{a_{2}}\log \biggl(\frac{v _{2}-\frac{b_{2}}{a_{2}}U}{V_{G}-\frac{b_{2}}{a_{2}}U} \biggr). $$

Substituting $v_{2}$ once again into (35), it follows that

36 $$ \mathcal{V}_{+}(\textbf{v}) = \frac{1}{a_{1}}\log \bigl(E(v_{1})\bigr)= \mathcal{V}_{-}(\textbf{v}). $$

However,

37 $$ \begin{aligned} \frac{\partial{\mathcal{V}_{+}}}{\partial{v_{2}}}_{\upharpoonleft \varGamma} \neq \frac{\partial{\mathcal{V}_{-}}}{\partial{v_{2}}}_{ \upharpoonleft\varGamma} = 0, \end{aligned} $$

so that $\mathcal{V}$ is not continuously differentiable.

All controlled trajectories in the synthesis are extremals, and away from *Γ*, the value function $\mathcal{V}$ satisfies the Hamilton–Jacobi–Bellman equation for the unconstrained optimal control problem

38 $$ \frac{\partial{\mathcal{V}(t, \textbf{v})}}{\partial t} + \frac{ \partial{\mathcal{V}(t, \textbf{v})}}{\partial\textbf{v}}f\bigl(t, \textbf{v}, u^{\ast}\bigr) + L\bigl(t, \textbf{v}, u^{\ast}\bigr) = 0, $$

where *L* is the Lagrangian of the problem (for time optimal control problems, as in our case, $L = 1$).

This conclusion follows from the method of characteristics (e.g., see [34]) but can also directly be verified using the explicit formulas derived above. That $\mathcal{V}$ is not differentiable on *Γ* does not invalidate the proof of optimality, although the standard optimality argument based on dynamic programming (e.g., [34], Theorem 5.2.1) does not apply. Here, we need to invoke regular synthesis constructions (see Appendix A.3) as they are described in [34, Sect. 6.3]. Since trajectories do not return from the state space constraint into the interior of the state space, these arguments could, for example, be undertaken by redefining the state space constraint as a second terminal manifold, along with a penalty term that gives the time along the boundary control until $v_{1}=V_{T}$. Alternatively, the constructions in [35], where a regular synthesis argument has been generalized to problems with order 1 state space constraints, could be modified to apply to cases where the state space constraint is active at the terminal time. Either way, straightforward modifications of regular synthesis type arguments give the optimality of the above field of extremals. □

#### Example 1

We demonstrate minimum spike time control in an example of (11) with the following parameters:

39 $$ \begin{aligned} &R_{1} = 0.5~{\mathrm{G \varOmega}},\qquad R_{2} = 0.33~{\mathrm{G\varOmega}}, \\ &C_{1} = 300~{\mathrm{pF}}, \qquad C_{2} = 300~{\mathrm{pF}}, \\ &V_{T} = 30~{\mathrm{mV}},\qquad V _{G} = 27~{\mathrm{mV}} \\ &U = 2.5~{\mathrm{nA}},\qquad \beta_{1} = 1, \qquad \beta_{2}= 1.2. \end{aligned} $$

**Fig. 3:**
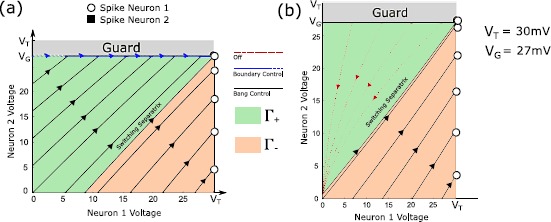
**(a)** State trajectories for selective spiking of Neuron 1 under Case 1 for several initial conditions. Trajectories either reach threshold under maximal input, or reach the guard under maximal input and then follow the boundary under a lower constant input until Neuron 1 reaches threshold. **(b)** State trajectories for selective spiking under Case 2 for several initial conditions. For those trajectories that do not reach Neuron 1 threshold (before hitting the guard) under maximal input, the input is zero until the trajectory decays to the switching separatrix, and then bangs high until Neuron 1 spikes.

Note that these are idealized parameters used for illustrative purposes only, although with biologically plausible units. Here, the condition $\gamma_{1}>\frac{V_{T}}{V_{G}}$ is satisfied, and we can apply the above proposition to induce a spike in Neuron 1 in minimal time. Figure 3(a) shows the state space under this construction.

### Selective Spiking, Case 2: $\gamma_{1}\leq\frac{V _{T}}{V_{G}}$

We now consider the case of eliciting a spike in Neuron 1 when $\gamma_{1}\leq\frac{V_{T}}{V_{G}}$.

We showed in the previous section that for Case 1, a control solution always exists. It will turn out that not all parameters allow a solution in Case 2, so this case reveals the conditions for pairwise feasibility of sequences while providing the minimum time spiking solution when it exists.

We might expect the solution in Case 2 to be qualitatively similar to Case 1, but in fact there are no longer increasing trajectories that ride along the guard boundary: under the boundary control ($u_{\mathrm{arc}} = \frac{a_{2}V_{G}}{b_{2}}$), we find $\dot{v}_{1}<0$ at $v_{1}=V_{T}$, that is, along the guard, $v_{1}(t)$ does not rise beyond a certain limit and fails to reach the threshold $V_{T}$. Instead, we have the following:

#### Proposition 2

*Consider the two*-*neuron network* (11), *where* $\gamma_{1}\leq\frac{V_{T}}{V_{G}}$. *Assume that the set of admissible controls is a box constraint* $\mathcal{U}=[0,U]$. *The time optimal control* $u^{*} \in\mathcal{U}$ *for the selective spiking problem P*1 *for Neuron *1, *if such a solution exists*, *is*

40 $$ u^{\ast} = \textstyle\begin{cases} 0 & \textit{for }\mathbf{v} \in\varGamma_{+} , \\ U & \textit{for }\mathbf{v} \in\varGamma_{-}, \end{cases} $$

*with* $\varGamma_{\pm}$ *defined as before*.

#### Proof

We follow a similar analysis to the previous case, but identify the differences in the optimal control structure from the solution in Sect. 3.1. Again, our approach is to define a synthesis of extremal controlled trajectories, prove their optimality, and finally give conditions for the existence of a solution for all $\textbf{v} \in\mathcal{G}$.

*Synthesis Construction.* The Hamiltonian and multiplier are similar to (18) and (20). The minimum condition similarly results in (21) with the conclusion that the optimal control is simply BANG at $u^{\ast}(t)=U$ for trajectories that do not hit the guard under this control. Similarly to (28), there again exists a curve *Γ* that separates such initial conditions from those requiring switching, given by (29). Note that there is no boundary segment in this case as $u_{\mathrm{arc}}$ cannot drive the voltage of Neuron 1 up to threshold along the state constraint boundary (see Appendix A.2), and thus we are led to consider controls only of the form

41 $$ u = \textstyle\begin{cases} 0 &\text{for $t < \hat{t}$ where $v_{1}(\hat{t}) < V_{T}$}, \\ U &\text{for $\hat{t} \leq t \leq\tau$}, \end{cases} $$

in the interior of $\mathcal{G}$, and $\hat{t}=0$ is allowed. *This concludes the synthesis construction.*

*Proof of Optimality.* The value function for the region $\varGamma_{-}$ equals the time taken by Neuron 1 to reach the threshold $V_{T}$ under the constant control *U* and takes the same form as (31). For $\textbf{v}\in\varGamma_{+}$, the value function is calculated assuming that the control is turned off for an interval $[0, \hat{t}]$, during which the system decays from the initial condition $\textbf{v}(0)=[v_{1} \ v_{2}]^{T}$ to a point $\textbf{v}( \hat{t}) = [\hat{v}_{1} \ \hat{v}_{2}]^{T}$ on the curve *Γ*. At this time the control switches to the maximum value *U*, and the corresponding trajectory follows the curve until the terminal condition $\textbf{v}(\tau)=[V_{T} \ V_{G}]^{T}$ is reached. This gives

42 $$ \mathcal{V}_{+}(\textbf{v}) = \hat{t}+t_{\mathrm{th}} = \frac{1}{a_{1}}\log \biggl(\frac{v_{1}}{\hat{v}_{1}} \biggr) - \frac{1}{a_{1}} \log\bigl(E( \hat{v}_{1})\bigr), $$

where

43 $$ \begin{aligned} \hat{v}_{2} = \biggl( \frac{\hat{v}_{1}}{v_{1}} \biggr)^{\frac{a_{2}}{a _{1}}}v_{2} \quad \text{and}\quad \biggl(E(\hat{v}_{1})\frac{\hat{v}_{1}}{v_{1}} \biggr)^{\frac{a _{2}}{a_{1}}}v_{2}+ \frac{b_{2}}{a_{2}}U \bigl(1 - E(\hat{v}_{1})^{\frac{a _{2}}{a_{1}}} \bigr) - V_{G} =0 \end{aligned} $$

using the fact that $[\hat{v}_{1}\ \hat{v}_{2}]^{T}$ lies on *Γ*. Here we cannot get an explicit expression for $\mathcal{V} _{+}$ in terms of the initial condition $[v_{1} \ v_{2}]^{T}$ because of the transcendental form of (43).

Note that, for this synthesis, the state space constraint does not become active. It is clear from the construction that the corresponding values satisfy the Hamilton–Jacobi–Bellman equation away from *Γ*. However, this problem is nonstandard in that the value function may no longer be continuous on *Γ*, with the only exception at $v_{1} = 0$, that is,

44 $$ \mathcal{V}_{+}(\textbf{v}) = \mathcal{V}_{-}( \textbf{v}) \quad \text{for } \textbf{v} \in\varGamma\text{ such that } v_{1} =0. $$

In general, there may exist a unique point on the curve *Γ* (in our problem with $u = 0$) where the vector field $\dot{\textbf{v}}=A \textbf{v}$ is tangent to *Γ* while pointing in the opposite direction. As a result, $\dot{\textbf{v}}=A\textbf{v}$ points into the region $\varGamma_{+}$ and into the region $\varGamma_{-}$, above and below this point, respectively. This generates a loss of small-time local controllability that causes the value function to become discontinuous along *Γ* above this point. For, if the initial condition lies to the right of $\varGamma_{+}$ above this point, then optimal trajectories must decay below the point in order to reach the terminal manifold. We see this in Fig. 3(b), where the OFF segment in the extremal cannot simply converge to the separatrix *Γ*, no matter how close it is to *Γ*. This issue of controllability makes the value function discontinuous. The value is still lower semicontinuous on the full state space. In fact, the value of this synthesis satisfies Sussmann’s weak continuity requirement [34, Definition 6.3.3]. Although the discontinuity of the value impedes on the application of most HJB-type sufficient conditions for optimality, this is not the case for regular synthesis-type constructions (see Appendix A.3), and the optimality of the synthesis follows from Theorem 6.3.3 in [34].

*Existence of Solution.* However, the control approach in (41) will fail if trajectories starting in $\varGamma_{+}$ do not in fact hit the separatrix at some time during the initial off-control. A necessary and sufficient condition for trajectories to hit the separatrix is that *Γ* intersects the positive $v_{2}$ axis. When this condition holds and $\textbf{v}(0)$ lies above *Γ*, then there must be a time *t̂* where the trajectory hits *Γ* under $u=0$. Conversely, suppose *Γ* does not intersect the positive $v_{2}$ axis. The slope of *Γ*, considering $v_{2}$ as a function of $v_{1}$, must be less than the slope of the decaying trajectory for there to be an intersection (ignoring the degenerate parameter choice for which tangency is possible). Taking the ratios $\dot{v}_{2}/\dot{v}_{1}$ for $u=0$ and $u=U$ (recalling that *Γ* is itself a solution with maximal input) and rearranging the result show that the slope condition can be met only if $v_{2}>\gamma _{1} v_{1}$. However, by our assumption $\gamma_{1}\le V_{T}/V_{G}$, no point on *Γ* meets this inequality (the curve lies entirely below the line from the origin to $[V_{T} \ V_{G}]^{T}$). In fact, since $\dot{v}_{i}$, $i =1,2$, is monotonic in *u*, it follows that there is no admissible control that can push a solution across *Γ*, so that the latter serves as a barrier to Neuron 1’s threshold for all initial conditions in $\varGamma_{+}$ (at least, without first crossing the Neuron 2 guard). So in this case, selective spiking of Neuron 1 is not possible.

Thus, the condition for the existence of a time-optimal solution for selective spiking of Neuron 1 is that the $v_{2}$ intercept of *Γ* is positive, which occurs when

45 $$ \biggl( 1-\frac{a_{1}V_{T}}{b_{1}U} \biggr) ^{a_{2}} > \biggl( 1- \frac{a _{2}V_{G}}{b_{2}U} \biggr) ^{a_{1}}. $$

 □

#### Example 2

We use the same parameter values as in (39) but swap the roles of Neuron 1 and Neuron 2, that is,

46 $$ \begin{aligned} &R_{2} = 0.5~{\mathrm{G \varOmega}}, \qquad R_{1} = 0.33~{\mathrm{G\varOmega}}, \\ &C_{2} = 300~{\mathrm{pF}}, \qquad C_{1} = 300~{\mathrm{pF}}, \\ &V_{T} = 30~{\mathrm{mV}}, \qquad V _{G} = 27~{\mathrm{mV}}, \\ &U = 2.5~{\mathrm{nA}},\qquad \beta_{2} = 1, \qquad \beta_{1}= 1.2. \end{aligned} $$

Now, $\gamma_{1} \leq V_{T}/V_{G}$. Moreover, condition (45) holds, so that the switching separatrix intersects the positive $v_{2}$ axis. Thus a time-optimal solution for selectively spiking Neuron 1 always exists. Figure 3(b) shows example trajectories.

### Geometric Interpretation of Cases and Pairwise Feasibility

Thus far in our discussion we assume, without loss of generality, that a selective spike is desired in Neuron 1. Now for pairwise feasibility, that is, to analyze when time-optimal selective spiking of either neuron is possible (from any initial condition), both neurons must be associated with either Case 1 or Case 2. To do this, we introduce

47 $$ \gamma_{2} = \frac{b_{2}a_{1}}{b_{1}a_{2}}= \frac{1}{\gamma_{1}}. $$

We associate Neuron 1 with $\gamma_{1}$ and Neuron 2 with $\gamma_{2}$ to determine the case (Sects. 3.1 and 3.2) to which these neurons belong. We say Neuron 1 is Case 1 or 2 when $\gamma_{1} > \frac{V_{T}}{V_{G}}$ or $\gamma_{1} \leq\frac{V_{T}}{V_{G}}$, respectively, and similarly for Neuron 2 with the same inequality relation on $\gamma_{2}$. Since we have $V_{T} > V_{G}$, this allows for three possible scenarios,

1. $\gamma_{1} > \frac{V_{T}}{V_{G}}$, $\gamma_{2} < \frac{V_{T}}{V_{G}}$: Neuron 1 is Case 1, and with $\gamma_{2}$ being the reciprocal of $\gamma_{1}$, we have Neuron 2 is Case 2.
2. $\gamma_{1} < \frac{V_{T}}{V_{G}}$, $\gamma_{2} > \frac{V_{T}}{V_{G}}$: Neuron 1 is Case 2 and Neuron 2 is Case 1, and the structure of the solution is identical to the previous scenario.
3. $\gamma_{1} \leq\frac{V_{T}}{V_{G}}$, $\gamma_{2} \leq \frac{V_{T}}{V _{G}}$: Both Neurons are Case 2, and this happens when $\frac{V_{G}}{V _{T}} \leq\gamma_{1,2} \leq\frac{V_{T}}{V_{G}}$.

As we will show in the following sections, for one of the neurons belonging to Case 1, pairwise selective spiking can be accomplished. However, if $\gamma_{1,2} \leq\frac{V_{T}}{V_{G}}$, that is, both neurons are Case 2, a solution may not exist, and pairwise feasibility is not guaranteed.

To provide an additional geometric interpretation (see Appendix A.2) of these conditions, we introduce the quasi-static equilibrium line

48 $$ \textbf{v}(\infty) := \bigl\{ (v_{1}, v_{2}) | b_{2}a_{1}v_{1} = b_{1}a _{2}v_{2}\bigr\} , $$

which defines the set of points for which $\dot{\textbf{v}}= \textbf{0}$ (for each $u \in\mathcal{U}$).

In a pair of neurons, the following two possible parameterization scenarios can be encountered.

#### Neuron 1 and 2 Correspond to Different Cases

Here we discuss the pairwise feasibility for when Neuron 1 is Case 1 and Neuron 2 is Case 2. It is important to note that the result extends to the reverse scenario, that is, Neuron 1 is Case 2 and Neuron 2 is Case 1.

Here, the line of quasi-static equilibrium in (48) intersects the line $v_{1}=V_{T}$ before it intersects $v_{2}=V_{G}$. Thus, Neuron 1 can always increase along the Neuron 2 guard boundary. Conversely, Neuron 2 cannot increase along the Neuron 1 guard beyond the point of intersection between $\textbf{v}(\infty)$ and $v_{1}=V_{G}$. As we showed before, in this case, selective spiking of Neuron 1 is always possible. Thus, pairwise feasibility reduces to condition (45) modulo a swapping of labels. Specifically, we have the following:

##### Lemma 1

*Consider the two*-*neuron network* (11), *where Neuron *1 *satisfies Case* 1, *and Neuron *2 *satisfies Case* 2. *Then*, *the network is pairwise feasible if and only if*

49 $$ \biggl( 1-\frac{a_{2}V_{T}}{b_{2}U} \biggr) ^{a_{1}} \geq \biggl( 1-\frac{a _{1}V_{G}}{b_{1}U} \biggr) ^{a_{2}}. $$

##### Proof

The proof follows immediately from Proposition 2 and (45), with a swapping of labels.

Thus, it follows that if (49) does not hold, a time-optimal solution for Neuron 2 does not exist (for all initial conditions), and thus the neurons are not pairwise feasible. □

#### Neuron 1 is Case 2; Neuron 2 is Case 2

If both neurons are Case 2, then pairwise feasibility would necessitate (49) holding to within a swapping of labels (i.e., so that either neuron can be selectively spiked). Clearly, this is impossible (see Appendix A.2) except for the limiting case where $V_{G}=V_{T}$, that is, the neurons are not guarded. In such a scenario, the optimal solution may produce simultaneous spiking of both neurons depending on the initial condition.

## Minimum Time Sequence Control

We now use the above results to analyze longer pairwise spiking sequences $\varSigma_{S}$ to solve the problem *P2*. Based on the results of the previous section for pairwise feasibility, that is, to allow all possible spike sequences for two neurons, we make the following assumption hereon.

### Assumption 1

The pair of neurons are parameterized so that Neuron 1 satisfies Case 1, Neuron 2 satisfies Case 2, and Lemma 1 holds.

This assumption ensures that the selective spiking solutions for the two neurons are given by Proposition 1 and 2, respectively.

We now analyze all the possible length 2 sequences, that is, $[1,1]$, $[1,2]$, $[2,1]$, and $[2,2]$, and recognize how we can use the basic characterizations developed in Sect. 3.1 and 3.2 to synthesize a time-optimal strategy for these sequences. We employ a dynamic programming approach where, using the time-optimal solution for the second spike in neuron *i*, we define a terminal cost and then solve the resulting optimal control problem for the first spike in neuron *j*, $i,j\in\{1,2\}$. Whereas the optimal synthesis for some of these sequences can be generalized from the solution of *P1*, we shall see that for the target sequence $[2,1]$, no time-optimal control solution may exist.

### Synthesis of All-2 Spike Sequences

Without loss of generality, consider the spike sequence $\varSigma_{S} = [1, 1]$ that we want to achieve in minimum time. We will use the concept of dynamic programming to solve the following problem:

50 $$ \begin{aligned} \text{min} \quad \mathbb{J}(u)& =\int_{0}^{\tau_{1}}\mathrm{d}t +\int_{\tau_{1}}^{\tau_{2}}\mathrm{d}t \\ \text{s.t.} \quad \dot{\textbf{v}}&=f(\textbf{v}, u) = A\textbf{v}+bu, \\ 0&\leq u(t) \leq U, \\ v_{1}(\tau_{1}) &= V_{T}, \qquad v_{1}\bigl({\tau_{1}}^{+}\bigr) = 0, \\ v_{1}(\tau_{2}) &= V_{T},\qquad v_{2}(t) \leq V_{G} \quad \text{for } t \in[\tau_{1}, \tau_{2}]. \end{aligned} $$

We will start from the last spike, Neuron 1, for this example and solve the minimum time problem *P1* for all the initial condition for Neuron 2, namely $v_{2} \in[0, V_{G}]$, $v_{1} = 0$, and use the solution of *P1* as the terminal cost $\varphi(\textbf {v}(\tau _{1}))$ for the previous spike, Neuron 1 again, in our case. So we will solve the following optimal control problem:

51 $$ \begin{aligned} \text{min}\quad \mathbb{J}(u) &= \int_{0}^{{\tau_{1}}}\mathrm{d}t + \varphi \bigl(v_{2}(\tau_{1})\bigr) \\ \text{s.t.} \quad \dot{\textbf{v}}&=f( \textbf{v}, u) = A\textbf{v}+bu, \\ 0&\leq u(t) \leq U, \\ v _{1}({\tau_{1}}) &= V_{T},\qquad v_{2}(t) \leq V_{G} \quad \text{for } t \in[0, { \tau_{1}}]. \end{aligned} $$

Now we will seek synthesis for all possible two spike sequences using (51).

#### Spike Sequence $[1,1]$

**Fig. 4:**
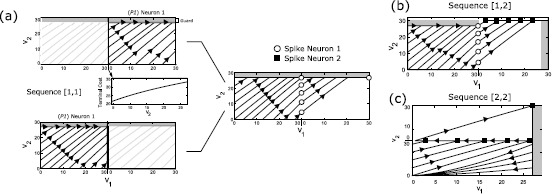
Optimal Synthesis for Sequences $[1,1]$, $[1,2]$ and $[2,2]$ is shown in **(a) (b) (c)** for the nominal parameters (39). In these depictions, the state space is repeated to indicate the reset condition. **(a)** Synthesis for $[1,1]$, showing both parts of the dynamic programming. The terminal cost is increasing and differentiable. The optimal trajectories from several initial conditions are shown. **(b)** Optimal trajectories for sequence $[1,2]$. **(c)** Optimal trajectories for sequence $[2,2]$. In this case, all initial conditions collapse onto a single manifold associated with the second spike.

The optimal synthesis for the sequence $\varSigma_{S}=[1,1]$ is given in Fig. 4(a). We highlight the solution of *P1* for Neuron 1 on the top left, the terminal cost $\varphi(v_{2}({\tau_{1}}))$ in the middle, and in the bottom, we show the solution of (51). On the right, we construct the complete synthesis for the whole sequence.

Given an arbitrary initial condition $[v_{1}\ v_{2}]^{T}$, the time-optimal solution of the first part without any terminal cost (i.e., $\varphi(v_{2}(\tau_{1})) \equiv0$, given by Proposition 1) has the property that, among all admissible controls, it leads to the smallest possible value for the terminal state $v_{2}({\tau_{1}})$. Since the function $\varphi(v_{2}({\tau_{1}}))$ is strictly increasing, this is then also the optimal solution for the combined problem and thus allows us to simply concatenate two solutions of *P1* for Neuron 1. Overall, the optimal control is simply given by the BANG control *U* until $v_{2}$ reaches the guard, after which the boundary control is used exactly as in the single spike problem.

#### Spike Sequence $[1,2]$

However, such monotonicity arguments do not work in the other cases. Figure 4(b) shows the synthesis of optimal controlled trajectories for the sequence $\varSigma_{S}=[1,2]$. The terminal cost $\varphi(v_{2}( {\tau_{1}}))$ is calculated as the value function from the solution of *P1* for Neuron 2 and is a strictly decreasing function of $v_{2}$ (since the higher the voltage $v_{2}$, the lower the time to induce a spike in Neuron 2). Thus, in principle, it might be possible for the solution of the first part to deviate from the solution of *P1* for Neuron 1 if the loss in doing so would be made up by the gain in the penalty function $\varphi(v_{2}(\tau_{1}))$ at the terminal point. Consider the switching function

52 $$ \varPhi(t) = \lambda_{1} b_{1} + \lambda_{2} b_{2}. $$

If there is a switching at $t = \hat{t}$, then we have

53 $$ \begin{aligned} \varPhi(\hat{t})& =\lambda_{1}(\hat{t}) b_{1} + \lambda_{2}(\hat{t}) b _{2} = 0, \\ \lambda_{1}(\hat{t}) b_{1} &= -\lambda_{2}( \hat{t}) b_{2}. \end{aligned} $$

Also, for a switching structure OFF-BANG, we must have

54 $$ \dot{\varPhi}(\hat{t}) < 0. $$

Now we use (53) for computing the derivative of the switching function

55 $$ \dot{\varPhi}(\hat{t}) = \lambda_{2}( \hat{t})b_{2}(a_{2}-a_{1}). $$

From the nontriviality [34, Sect. 2.2] and transversality conditions we have

56 $$ \lambda_{2}({\tau_{1}}) = \frac{\partial{\varphi(v_{2}({\tau_{1}}))}}{ \partial{v_{2}}} < 0, $$

since the terminal cost is a decreasing function of $v_{2}$. Also, we have previously derived that the adjoint variables are solutions of linear homogeneous differential equations that do not change sign in $t \in[0, \tau_{1}]$. So we have $\lambda_{2}(\hat{t}) < 0$ as well. Using these and assuming that $a_{2} < a_{1}$, from (55) we get

57 $$ \dot{\varPhi}(\hat{t}) > 0. $$

This violates the necessary condition in (54) for an OFF-BANG switching. Note that for the case $a_{1} < a_{2}$, OFF-BANG switching cannot be ruled out using this argument, and the synthesis has to be constructed by direct computation. In our example with the parameters from (39), it turns out that the optimal solution is simply BANG/BANG-BOUNDARY (17), that is, the terminal cost $\varphi(v_{2})$ has no effect on the solution of (51). Thus the time optimal synthesis for $\varSigma_{S}=[1,2]$ is a combination of the individual synthesis for Neurons 1 and 2.

#### Spike Sequence $[2,2]$

Similar controllability properties also allow us to give a short solution for the sequence $\varSigma_{S}=[2,2]$. The optimal synthesis is shown in Fig. 4(c). In this case, the terminal cost $\varphi(v_{1}({\tau_{1}}))$ is a function of $v_{1}$, and it is also strictly increasing in $v_{1}$ (since the higher the value of $v_{1}$, the higher the time to ensure selective spiking in Neuron 2). From the analysis of transversality condition and the switching function like in the previous sequence (54) we can show that OFF-BANG is optimal for the first spike in Neuron 2 with $a_{1} < a_{2}$ and suboptimal for $a_{2} < a_{1}$ if there exists a switching. Indeed, for the first Neuron 2 spike and initial conditions under the separatrix, the optimal control is OFF-BANG. But for initial conditions on the $v_{2}$ axis, the optimal control is simply BANG. In the example, the overall construction is achieved by concatenating the solutions of *P1* for Neuron 2 vertically. Since Neuron 2 is reset to 0 after firing, the initial condition for the second problem is given by $[v_{1}(\tau_{1}) \ 0]^{T}$.

#### Spike Sequence $[2,1]$

##### Proposition 3

*Under Assumption* 1, *no time optimal control solution exists in general for a target sequence* $\varSigma_{S}$ *containing the subsequence* $[2,1]$.

##### Proof

**Fig. 5:**
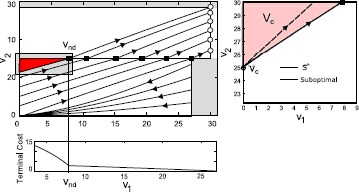
A possible suboptimal synthesis is shown for the sequence $\varSigma_{S} = [2,1]$. Note that the value function for the last spike, i.e. Neuron 1, plotted in the bottom panel, is not differentiable with respect to $v_{1}$. This is added as the terminal cost for the optimum control problem for the first spike in Neuron 2. In the right panel, the actual optimal solution and a constant control suboptimal synthesis proposed in (67) is shown.

The synthesis is more involved for this sequence. The terminal cost for the first Neuron 2 spike is the value function from (30) with $v_{2} = 0$, that is,

58 $$ \varphi\bigl(v_{1}(\tau_{1})\bigr)= \mathcal{V}(\textbf{v})|_{v_{2}=0}, $$

which is a decreasing function in $v_{1}$, and $\varphi(v_{1}(\tau _{1}))$ is not differentiable with respect to $v_{1}$ for some $v_{1} = v_{\mathrm{nd}}$ where $v_{\mathrm{nd}} \in[0, V_{G}]$ (as shown in the bottom left of Fig. 5). Note that for any initial condition at the origin or on the $v_{1}$ axis to the left of the separatrix, OFF-BANG cannot lead to optimality, and for those cases, the extremals will be generated by $u^{\ast}(t) = U$ for all $t \in[0, {\tau_{1}}]$. Also, to the right of the separatrix OFF-BANG will be the optimal policy as it is the only viable option in the presence of state constraints. So we can conclude that if there is indeed a switching to the left of the separatrix, then there must exist $v_{s} \in(0, V _{G}]$ such that for $\textbf{v}(0)=\{(v_{1}, v_{2}) : v_{1} = 0, v _{2} \in(v_{s}, V_{G})\}$, the optimal policy will be OFF-BANG, whereas for $\textbf{v}(0)=\{(v_{1}, v_{2}) : v_{1} = 0, v_{2} \in[0, v_{s}] \}$, the optimal control is BANG. Now we will calculate this voltage $v_{s}$, which acts as an onset for the change in optimal policy. Considering the switching at $t = \hat{t}$, we have $v_{2}(\hat{t}) = v_{s}$ and

59 $$ \varPhi(\hat{t}) = \lambda_{1}(\hat{t}) b_{1} + \lambda_{2}(\hat{t}) b _{2} = 0. $$

Since the Hamiltonian vanishes identically for our problem, we get

60 $$ \mathcal{H}(\hat{t}) = 1- a_{2} v_{s} \lambda_{2}(\hat{t}) = 0. $$

Also, from the transversality condition with $\lambda_{0} =1$ we have

61 $$ \lambda_{1}(\tau_{1}) = \frac{\partial{\varphi(v_{1}(\tau_{1}))}}{ \partial{v_{1}}}, $$

which is known. Since we reach the threshold $V_{T}$ from $v_{s}$ using the BANG control, from (26) we have

62 $$ \tau_{1} - \hat{t} = \frac{1}{a_{2}}\log \biggl( \frac{v_{s}-\frac{b _{2}}{a_{2}}U}{V_{T}-\frac{b_{2}}{a_{2}}U} \biggr). $$

Using the fact that the adjoint variables satisfy linear homogeneous differential equations, we can write

63 $$ \lambda_{1}(\hat{t}) = \frac{\partial{\varphi(v_{1}(\tau_{1}))}}{ \partial{v_{1}}} \biggl( \frac{v_{s} - \frac {b_{2}}{a_{2}}U}{V_{T}-\frac{b _{2}}{a_{2}}U} \biggr)^{-\frac{a_{1}}{a_{2}}}. $$

From (59)–(63) we can solve for $v_{s}$ with

64 $$ 1+\frac{a_{2} v_{s} b_{1}}{b_{2}}\frac{\partial{\varphi(v_{1}(\tau _{1}))}}{\partial{v_{1}}} \biggl(\frac{v_{s} - \frac{b_{2}}{a_{2}}U}{V _{T}-\frac{b_{2}}{a_{2}}U} \biggr)^{-\frac{a_{1}}{a_{2}}} = 0. $$

If such $v_{s}$ exists, then the construction may be much more complicated with the possible presence of a “cut-locus” type phenomenon, and we leave a detailed analysis of such a problem for future work. In our case, the terminal cost decreases with a rapid rate for $v_{1} \in[0, v_{nd}]$ and abruptly changes to a much smaller slope for $v_{1} \in(v_{\mathrm{nd}}, V_{G}]$ (see Fig. 5) due to the nature of the value functions on either side of separatrix $\mathcal{V}{-}$, $\mathcal{V}{+}$ in (31) and (32). This results in a field of extremals trying to converge to the point $v_{\mathrm{nd}}$, even when the monotonicity of the value function is not affected by the loss of differentiability (see top left in Fig. 5). We calculate the set of initial conditions for which this point can be attained, specifically $\textbf{v}_{c} = \{(v_{1}, v_{2}): v_{1} = 0, v_{2} \in[v_{c}, V_{G}]\}$, where $v_{c}$ denotes the highest point on $v_{2}$ axis from which $[V_{T} \ v_{\mathrm{nd}}]^{T}$ can be reached via BANG control. This voltage $v_{c}$ and the set $\textbf{v}_{c}$ are shown in the right panel of Fig. 5. Now, the optimal control problem for $\textbf{v}(0) \in\textbf{v}_{c}$ simply reduces to

65 $$ \begin{aligned} \min\quad \mathbb{J}(u) &=\int_{0}^{{\tau_{1}}}\mathrm{d}t \\ \text{s.t.} \quad u(t) &\in\mathcal{U} \end{aligned} $$

with the terminal constraint $\textbf{v}({\tau_{1}}) = [v_{\mathrm{nd}}\ V_{T}]^{T}$ and state constraints $v_{1}(t) \leq V_{G}$, $v_{2}(t) \leq V_{T}$. This is similar to the selective spiking problem of Neuron 1, and indeed the best control is a combination of BANG and boundary control as in (17),

66 $$ u^{\ast} = \textstyle\begin{cases} U &\text{for } t \leq t_{c} \text{ where } v_{2}(t_{c}) = V_{T}, \\ u_{\mathrm{arc}}&\text{for } t_{c} < t \leq{\tau_{1}} \text{ where } v_{1}({\tau_{1}}) = v_{\mathrm{nd}}. \end{cases} $$

But this implies that Neuron 2 maintains the voltage $(V_{T})$, even after the spike is emitted, which violates our assumption that the neurons are reset instantaneously after reaching $V_{T}$, as described in (4). So the synthesis $\mathcal{S}^{\ast}$ corresponding to (66) is excluded from the admissible set of extremals purely out of the physical constraints imposed on the system. This resembles the classical problem of finding surfaces of minimum revolution [34], where the Goldschmidt extremal cannot be attained because of the *C* ^1^ assumption on the extremals. Thus, any synthesis $\mathcal{S}$ for (65) will be suboptimal to $\mathcal{S} ^{\ast}$. For simplicity, we have picked a synthesis such that

67 $$ u_{\mathrm{sub}} = u\bigl(\textbf{v}(0)\bigr) \quad \text{for } t\in[0, {\tau_{1}}], $$

that is, a constant control that varies depending on the initial condition shown in Fig. 5. For the set of initial conditions

68 $$\begin{aligned} \textbf{v}(0) &= \bigl\{ (v_{1}, v_{2} ) : v_{1} = 0, 0 \leq v_{2} < v _{c}\bigr\} \\ &\quad {}\cup\bigl\{ (v_{1}, v_{2}) : 0 \leq v_{1} < V_{G}, v_{2} = 0\bigr\} , \end{aligned}$$

the optimal synthesis remains the same as the solution of *P1* for Neuron 2.

□

### Greedy Designs for Sequences with Arbitrary Length

From our analysis of the 2-spike sequences in the previous section, we can design the time optimal control for any $\varSigma_{S}$ of *M* spikes ($M \geq2$) without the subsequence $[2, 1]$. In addition, if we assume that $a_{2} < a_{1}$, then it can be shown using an inductive argument that the overall synthesis can be constructed from the solutions of individual selective spiking problems in Propositions 1 and 2.

In general, for a $\varSigma_{S}$ with the subsequence $[2,1]$, to illustrate the complexities of sequence control, it is instructive to consider the 4-spike sequence $\varSigma_{S} = [1,2,1,1]$. In this case, the target sequence contains a $[2,1]$ event, meaning that any solution will be suboptimal. In this case, a dynamic programming approach that interleaves the interpolation control (67) can yield such a solution. However, from a practical perspective, pursuing this design approach for long sequences is difficult as it requires computing the location of nondifferentiability in the value functions of all $[2,1]$ events.

Thus, we argue that, from a design perspective, a simple greedy approach, where we minimize the time for each spike in $\varSigma_{S}$ progressively, constitutes an acceptable and tractable approximation.

**Fig. 6:**
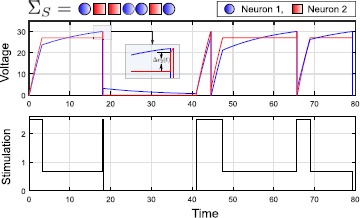
Simulation example of the greedy algorithm for *P2* for a target sequence $\varSigma_{S} = [1,2,2,1,1,2,1]$ with the nominal parameters in (39). The inset shows the synaptic contribution $\Delta v_{2}(t) =2\mbox{ mV}$, to Neuron 2 due to the first spike in Neuron 1.

In Fig. 6, we show the solution of the greedy controller for an arbitrary spike sequence $\varSigma_{S}$.

#### Decoupling the Network for Longer Sequences

In applying the greedy approach, it is important to note that the synaptic contribution from the spiking neuron can carry the voltage of the other neuron in the network over the synaptic guard $V_{G}$. Thus, we cannot readily apply the solution of *P1* for the following spike in the sequence (pattern), as the initial condition may violate the state constraint in (8) for *P1*. Here, we propose strategies to eventually utilize Propositions 1 and 2 for the greedy design.

1. First, if the initial condition after any spike in the sequence (pattern), at $t = \tau_{1}$, is not within the relevant state space $\mathcal{G}$, then we can apply $u =0$ until $t= t'$, $t' > \tau_{1}$, such that $\mathbf{v}(t') \in\mathcal{G}$. Then, we can apply the solution of *P1* to induce the target spike.
2. Alternatively, we can modify the guard $V_{G}$ of the nontarget neuron at each step of the greedy design, depending on the number of consecutive spikes in the target neuron in the sequence (pattern); for example, if $\varSigma_{S} = [1 , 1, 2, 2, 2]$, then we can set the guard voltage for Neuron 2 at $V_{G}(\sigma_{1}) < V_{T} - 2 \bar{\rho} _{\mathrm{syn}} $ for the first spike and $V_{G}(\sigma_{2}) < V_{T} - \bar{ \rho}_{\mathrm{syn}}$ for the second spike. Thus, the relevant state space for the first and second spikes will be modified to $\mathcal{G}( \sigma_{1}) = [0, V_{T}] \times[0, V_{G}(\sigma_{1})]$ and $\mathcal{G}(\sigma_{2}) = [0, V_{T}] \times[0 ,V_{G}(\sigma_{2})]$, respectively. This ensures that whatever the contribution is from the presynaptic neuron (in this case, Neuron 1), we start in the relevant state space for the next spike in the sequence (pattern). Once the target neuron changes to $\sigma_{3} = 2$, the guard voltage for Neuron 1 is determined by the number of consecutive spikes in Neuron 2 (3 in this example), that is, $V_{G}(\sigma_{3}) < V_{T} - 3 \bar{\rho} _{\mathrm{syn}}$ and so on. Note that by successively reducing the guard voltage, the selective spiking problem may become infeasible as discussed in Sect. 3.3.
3. Finally, we can combine the two approaches to develop an algorithm where we can use (2) until the problem is infeasible. At this point, we go back to (1) and add an off time before implementing the solution of *P1*.

**Fig. 7:**
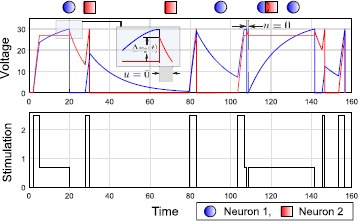
Simulation example of the greedy algorithm discussed in Sect. 5.2 for *P3* for a target pattern $\varSigma_{P} = [(1,20),(2, 30),(2, 70),(1,95),(1, 115),(2,120), (1, 130)]$ with the nominal parameters in (39). Similar to Fig. 6, we show the synaptic contribution $\Delta v_{2}(t) =2\mbox{ mV}$, to Neuron 2. We also explicitly indicate the off-time $(u=0)$ after the first (inset) and fourth spike in Neuron 1, as part of the decoupling strategy discussed in Sect. 4.2.1.

In our examples of sequence and pattern control, we have used the first approach in developing the greedy design (see Figs. 6 and 7).

## Fixed-Time Selective Spiking and Spike Patterns

We now move to the problem of controlling timed spike patterns, that is, *P3*. It is intuitive that a basic necessary condition in this case is that the desired spike time exceeds the minimum selective spiking time, that is, the solution to *P1*.

Specifically, suppose that we want to achieve the target pattern $\varSigma_{P} = [(1, t_{1})]$, that is, a spike in Neuron 1 at time $t_{1}$. The cost function in *P3* (14) reduces to

69 $$ \mathbb{J}(u) = \biggl( t_{1}- \int_{0}^{\tau_{1}}\mathrm{d}t \biggr) ^{2} $$

(subject to the selectivity constraint in (7)). Here, $\tau_{1}$ denotes the achieved spike time, and $\bar{\tau}_{1}$ is the solution of *P1* for an arbitrary initial condition $\mathbf{v}(0)$. If $\bar{\tau}_{1} \geq t_{1}$, then evidently that is our best option, and the solutions of (69) and *P1* are the same, that is, $\tau_{1} = \bar{\tau}_{1}$.

For the other case, $\bar{\tau}_{1} < t_{1}$, contingent on controllability, a control must exist such that $\tau_{1} = t_{1}$. If such a condition is met, then in general there may be multiple solutions to the pattern control problem.

Herein, we consider one simple strategy involving the introduction of an off time *t̂* to the optimal control solution of *P1* such that

70 $$ \hat{t} + \tau_{1}^{r} = t_{1}, $$

where $\tau_{1}^{r}$ is the solution of the time optimal control *P1*, for the initial condition $\textbf{v}(\hat{t})$.

We noted earlier that the initial conditions for the selective spiking problem nominally lie on either the $v_{1}$ or $v_{2}$ axis, under the assumption that one of the neurons has just produced a spike. In this case, feasibility of (70) reduces to understanding those initial conditions that generate specific values of $\tau_{1}^{r}$.

### Off-Time Insertion for Pattern Control

We characterize the relationship between $\tau_{1}^{r}$ and initial conditions via the notion of a *Λ*-controllable set.

#### Definition 3

*Λ*-Controllable set

Without loss of generality, the *Λ*-controllable set $\zeta(\varLambda)$ of Neuron 1 is the set of initial conditions from which the selective spiking of Neuron 1 in *P1* is achieved in time *Λ*, that is,

71 $$ \zeta_{1}(\varLambda) = \bigl\{ (v_{1}, v_{2}): \mathbf{v}(0) = [v_{1} \ v _{2}]^{T}, \nexists t < \varLambda \text{ s.t. } v_{1}(t) = V_{T}, v_{2}(t) \leq V_{G}\bigr\} . $$

The *Λ*-controllable sets for system (11) are provided in Appendix A.4. Since we are interested in initial conditions along the $v_{1}$ and $v_{2}$ axes, we consider the functions

72 $$ \begin{aligned} \omega_{1} &: \varLambda \rightarrow v_{1},\quad \text{such that } (v _{1},0) \in\zeta_{1}(\varLambda), \\ \omega_{2} &: \varLambda \rightarrow v_{2},\quad \text{such that } (0, v_{2}) \in\zeta_{1}( \varLambda), \end{aligned} $$

that is, the intersection of the *Λ*-controllable sets with the axes.

Earlier, we noted that the value function for the selective spiking of both neurons remains continuous on both the $v_{1}$ and $v_{2}$ axes (i.e., from (36) and (44)). This fact, together with the derivation of the *Λ*-controllable sets in the Appendix, allows us to conclude that the functions (72) are continuous in *Λ*.

Thus, we are able to ensure the existence of the *off-time pattern control* from (70), that is,

73 $$ {u^{p}} = \textstyle\begin{cases} 0 &\text{for $t \in[0 ,\hat{t}]$}, \\ {u^{*}} &\text{for $t \in(\hat{t},t_{1}]$}, \end{cases} $$

where $u^{*}$ comes from Proposition 1 or 2. The computation of the off-time *t̂* is obtained directly from the *Λ*-controllable sets and is provided in Appendix A.5. Thus, an overall pattern control strategy can be formulated as

74 $$ \varPi^{\ast} = \textstyle\begin{cases} u^{\ast} &\text{if $\bar{\tau}_{1} \geq t_{1}$}, \\ u^{p} &\text{if $\bar{\tau}_{1} < t_{1}$}. \end{cases} $$

### Greedy Designs for Control of Long Patterns

We now consider the synthesis and design of the general pattern control problem *P3*. To begin, we consider the dynamic programming strategy studied in (51) but for *P3*. It turns out that the same issues pertaining to nondifferentiability of the value function in *P2* persist in this case.

To illustrate this, consider the 2-spike target pattern $\varSigma_{P} = [(1, t_{1}), (1, t_{2})]$. Starting from the last spike $\sigma_{2} = 1$, we solve

75 $$ \mathbb{J}(u) = \biggl( (t_{2}-t_{1})- \int_{\tau_{1}}^{\tau_{2}} \mathrm{d}t \biggr) ^{2} $$

with the terminal and state constraints and use the value function of (75) as the terminal cost to the following optimal control problem:

76 $$ \mathbb{J}(u) = \biggl( t_{1}- \int_{0}^{\tau_{1}}\mathrm{d}t \biggr) ^{2} + \varphi\bigl(v_{2}(\tau_{1})\bigr). $$

Let us denote the solution of *P1* for the second spike from the initial condition $\mathbf{v}(0) = [0 \ v_{2}]^{T}$ by *τ̄*. Then, depending on $v_{2}$, the terminal cost in (76) takes the following form:

77 $$ \varphi(v_{2}) = \textstyle\begin{cases} 0 &\text{for $v_{2}$ s.t. $\bar{\tau} \leq(t_{2} - t_{1})$,} \\ ((t_{2}-t_{1}) - \bar{\tau})^{2} &\text{for $v_{2}$ s.t. $\bar{ \tau} > (t_{2} - t_{1})$}. \end{cases} $$

Thus, similar complications as referenced in Sect. 4.2 regarding nondifferentiability arise here, and once again we consider implementation of a straightforward greedy strategy for pattern control involving (74). In Fig. 7, we show an example of this greedy algorithm for an arbitrary pattern with the same spike sequence as in Fig. 6.

### Performance of Greedy Design Under Disturbance and Noise

In this section, we analyze the robustness of the greedy design when the coupled LIF network in (3) is subjected to noise and disturbances. Here we consider two types of uncertainties:

1. Incoming synaptic contributions of the pulse coupled form discussed in Sect. 2.2.2, from other neurons
2. Noise in the dynamics of the membrane voltage of the neurons in (3) (process noise) and in measurement of these voltages (measurement noise). Note that in implementing the greedy controller in (74), we repeatedly apply Propositions 1 and 2, which are feedback control, that is, measurement is implicit.

**Fig. 8:**
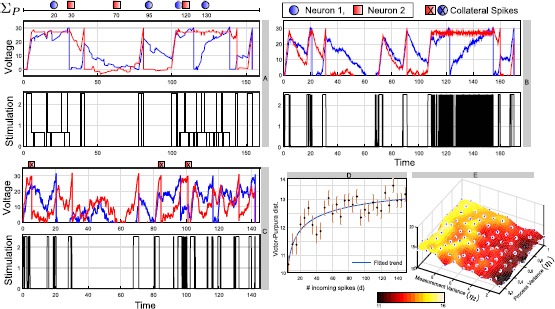
Induced voltage waveforms in the two neurons for $\varSigma_{P} = [(1,20),(2, 30), (2, 70),(1,95),(1, 115),(2,120), (1, 130)]$ using the greedy design and the control under incoming synapses **(A)** and process, measurement noise (**(B)** for higher variance and **(C)** for lower variance). **(D)** Performance analysis of the controller in terms of VP distance with parameters $q=1$, $k = 1.5$ against number of incoming spikes *d* as measure of disturbance. **(E)** Surface plot fitted to the simulation data of average VP distance (same *q*, *k*) vs the process and measurement noise variances, in the course of solving the pattern control problem for $\varSigma_{P}$ over different trials.

In Fig. 8(A), we show one realization of the voltage and control waveforms for $d = 150$ incoming spikes over the control horizon for the same $\varSigma_{P}$ used in the example of Fig. 6. To illustrate the effect of these disturbances on the control strategy, in Fig. 8(D), we plot the average Victor–Purpura (VP) distance [36, 37] between the achieved and target spike trains as we vary the number of incoming spikes *d* over 50 trials. In each trial, we randomly select the arrival times of the spikes, the contribution and target of the synapse between the two neuron indices. The VP metric is a measure of synchrony between two spike patterns that involves three basic operations: adding or deleting any spike with cost 1, moving any spike with cost *q* per unit time, and renaming any index of the spike with cost *k*. Here, a lower VP distance corresponds to better control performance. We observe that with higher disturbance, represented by *d*, the controller performs reasonably well with gradual degradation in the achieved patterns.

Next, we consider additive Gaussian noise both during the evolution of the membrane voltage and in measurement. Thus the linear model in (11) is modified to

78 $$ \begin{aligned} &\dot{\mathbf{v}}(t) = A\mathbf{v}(t) + b u(t) + \mathbf{w}(t), \\ & \mathbf{y}(t) = C\mathbf{v}(t) + \mathbf{z}(t), \end{aligned} $$

where the measurement vector **y** is a linear readout of the neuron voltages through a randomly selected matrix *C*, which is full rank, $\mathbf{w}(t)$ and $\mathbf{z}(t)$ follow multivariate Gaussian distributions with $\mathbf{w}(t) \sim\mathcal{N}(\mathbf{0}, W)$ and $\mathbf{v}(t) \sim\mathcal{N}(\mathbf{0}, Z)$, and *W* and *Z* are the constant covariance matrices of the forms $W = \eta_{1}^{2} \mathbb{I}$ and $Z = \eta_{2}^{2} \mathbb{I}$, $\mathbb{I}$ is the 2-dimensional identity matrix. Here, we compute the voltage estimates of the two neurons at each time step by means of a Kalman filter [38] and employ the feedback strategy in (74) based on these estimates. In Fig. 8 (B), (C), we plot the pattern control solutions for the same $\varSigma_{P}$ used in the example of Fig. 6 for smaller $(\eta_{1} = 0.1, \eta_{2} = 1)$ and higher $(\eta_{1} = 1, \eta_{2} = 10)$ process and measurement variance. We observe that controller’s ability to induce the target spike train is not compromised substantially, although with higher levels of noise, spurious spikes are generated, as indicated in panel (C). However, the noisy dynamics in (78) can result in a high frequency of switching in the control ((B), (C), bottom panel), especially during the boundary arc, that is, the nontarget neuron is to be held at guard $V_{G}$. Panel (E) shows the performance of the greedy design with respect to the average VP distance between $\varSigma_{P}$ and achieved patterns over 50 different trials, as we change the level of noise during the evolution and measurement phase.

## Conclusions

This paper has examined the problem of controlling timed spike patterns in pairs of Integrate and Fire neurons. Boundary-arc-type phenomena are shown to arise in this scenario due to state constraints imposed by both the selectivity criterion and spike generation mechanism. Formal analysis and synthesis is carried out to establish how the proposed solutions are geometrically disassociated in terms of their initial conditions. The developed solutions, which leverage the maximum principle and dynamic programming, are shown to be efficacious in controlling the LIF models.

Clearly, our results here are of theoretical nature. Although the control-theoretic features revealed are themselves interesting from a mathematical standpoint, they serve the broader purpose of establishing fundamental limits on the selective control of neurons with common inputs. The qualitative nature of the derived solutions (e.g., OFF-BANG, boundary-arc strategies) are already more complex than the fixed-amplitude square pulse designs currently used in practice. Given the massive growth in stimulation technology development, understanding these limits, even for a relatively simple model class, may provide insight into how experimentalists should tune their stimulation parameters for experimental objectives. For instance, our analytical conditions (e.g., $\gamma_{1} \gtrless\frac{V_{T}}{V_{G}}$) amount to a criteria on the amount of heterogeneity needed within a neuronal population in order to enable control. Without sufficient heterogeneity, it is simply impossible for a common input to “split” the spiking of neurons in a selective manner. Exploiting this heterogeneity is at the heart of the derived control solution (e.g., OFF-BANG solutions that leverage increased leak dynamics). These characterizations provide a baseline from which we plan to establish relaxations of the considered problems for larger neuronal populations in future work.

## Figures and Tables

**Fig. 9:**
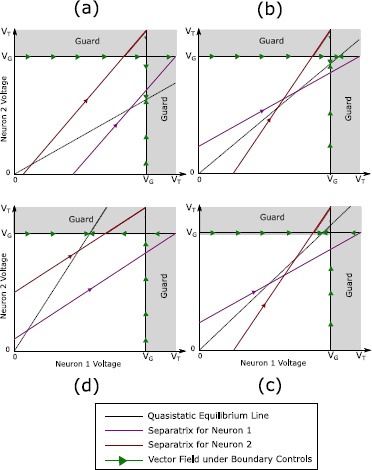
Representation of the solution space with respect to the quasistatic equilibrium line. **(a)** When Neuron 1 satisfies Case 1 i.e. $\gamma_{1} > {V_{T}}/{V_{G}}$, which implies $\gamma_{2} < {V_{T}}/{V_{G}}$, i.e. Neuron 2 satisfies Case 2. **(b)**, **(c)** The parameters of the neurons are such that $1 \leq\gamma_{1} \leq {V_{T}}/{V_{G}}$ and ${V_{G}}/{V_{T}} \leq\gamma_{1} \leq1$ respectively which implies both neurons are Case 2. Note that for these two scenarios, the selective spiking is not possible for both the neurons. **(d)** The parameters satisfy $\gamma_{2} > {V_{T}}/{V_{G}}$, i.e. Neuron 2 is Case 1 which implies Neuron 1 is Case 2, $\gamma_{1} < {V_{T}}/{V_{G}}$. For **(a)** and **(d)**, selective spiking is possible for both neurons.

## Appendix

### A.1 Derivation of Impulsive Synaptic Coupling Model

To derive (5), we start with a classical continuous-time model of synaptic dynamics [33] wherein, assuming that $V_{\mathrm {rest}}=0$ in (3), the membrane potential of each neuron evolves according to

79 $$ C\frac{{\mathrm{d}{v}(t)}}{{\mathrm{d}t}} = -\frac{{v}(t)}{R} + \beta u(t) + I_{\mathrm{syn}}(t) $$

with

80 $$ \begin{aligned} I_{\mathrm{syn}}(t) =g_{\mathrm{syn}}(t) \bigl(v(t) - E_{\mathrm{syn}}\bigr),\quad g_{\mathrm{syn}}(t) = \bar{g}_{s}\mathrm{e}^{-\frac{(t- t_{s})}{\kappa_{s}}} \mathcal{H}(t- t_{s}), \end{aligned} $$

where $t_{s}$ is the arrival time of a presynaptic action potential from the other neuron, $g_{\mathrm{syn}}$ is the synaptic conductance, $\mathcal{H}$ is a Heaviside step function, $\kappa_{s}$ is the time constant for the conductance, $\bar{g}_{s}$ is the maximum conductance for the synapse, and $E_{\mathrm{syn}}$ denotes the reversal potential. For selective spiking, we want the postsynaptic neuron to be protected from this incoming synapse with respect to the membrane potential.

In the typical case of an excitatory synapse, we have $E_{\mathrm{syn}} \approx0$, and the contribution from the spike in the presynaptic neuron becomes

81 $$ \Delta v(t) = \frac{1}{C} \int_{t_{s}}^{t}\bar{g}_{s} \mathrm{e}^{-\frac{(t- t_{s})}{ \kappa_{s}}}v(t)\,\mathrm{d}t. $$

Now, assuming a separation in time scale between the synaptic time constant $\kappa_{s}$ and the membrane time constant $\kappa_{m}$, that is, $\kappa_{s} \ll\kappa_{m}$, we can approximate the integral in (81) by keeping the voltage of the postsynaptic neuron constant at $v(t_{s})$ over the integration window. Using this, we have

82 $$ \Delta v(t) \leq\frac{1}{C} \int_{t_{s}}^{\infty}I_{\mathrm{syn}}(t) \,\mathrm{d}t = \frac{\bar{g}_{s}\kappa_{s}}{C}v(t_{s}) \leq\frac{ \bar{g}_{s}\kappa_{s}}{C}V_{T}. $$

So the effect of a synaptic event on the postsynaptic neuron can be crudely summarized as an almost instantaneous rise in voltage bounded by (82). Thus, model (5) approximates this effect with an impulsive synaptic action, where

83 $$ \Delta v(t) \equiv\rho_{\mathrm{syn}}(t) \leq \frac{\bar{g}_{s}\kappa_{s}}{C}V_{T} \equiv\bar{\rho}_{\mathrm{syn}}. $$

### A.2 Geometrical Aspects of Selective Spiking Solution

Here, we first discuss the role of $\gamma_{1}$ (15) in determining the two different selective spiking solutions presented in Sects. 3.1 and 3.2. We also geometrically show that pairwise feasibility is not achievable when both neurons are Case 2, as described in Sect. 3.3.2.

We first derive the equation for the line of quasistatic equilibrium defined in (48). This is the set of points in the phase plane for which $\dot{\textbf{v}}(u) = \mathbf{0}$ for any constant control $u \in\mathcal{U}$. Using this condition, we have

84 $$ \dot{v}_{1} = -a_{1}v_{1} + b_{1}u = \dot{v}_{2} = -a_{2}v_{2} + b _{2} u = 0. $$

Since *u* is a constant, we can eliminate *u* to get the equation for the quasistatic equilibrium

85 $$ \frac{v_{1}}{v_{2}} = \gamma_{1}, $$

where $\gamma_{1}= \frac{b_{1}a_{2}}{b_{2} a_{1}}$. Now the two different solutions presented in Propositions 1 and 2 are dependent on the existence of the boundary segment, that is, for a boundary control $u_{\mathrm{arc}}$ for which Neuron 2 is voltage invariant $(\dot{v}_{2}(u_{\mathrm{arc}}) = 0)$, regardless of whether the voltage of Neuron 1 increases. To satisfy this, we must have

86 $$ \dot{v}_{1}(u_{\mathrm{arc}})|_{v_{1} = V_{T}} > 0. $$

We can answer this question from the analysis on the quasistatic equilibrium line as $u_{\mathrm{arc}}$ is constant. If the line intersects $v_{1} = V_{T}$ before $v_{2} = V_{G}$, from (85) we have (see Fig. 9(a))

87 $$ \gamma_{1} > \frac{V_{T}}{V_{G}}. $$

Using this, we can calculate the direction of vector field at $v_{1} = V_{T}$ in (86):

88 $$\begin{aligned} \dot{v}_{1}(u_{\mathrm{arc}})|_{v_{1} = V_{T}}&= -a_{1} V_{T} + b_{1} u _{\mathrm{arc}}=-a_{1} V_{T} + b_{1} \frac{a_{2} V_{G}}{b_{2}} \\ &= a _{1} V_{G} \biggl(\frac{b_{1} a_{2}}{b_{2} a_{1}} -\frac{V_{T}}{V_{G}} \biggr)= a_{1} V_{G} \biggl(\gamma_{1} - \frac{V_{T}}{V_{G}} \biggr) > 0. \end{aligned}$$

We show this in Fig. 9(a), where the quasistatic equilibrium intersects $v_{2} = V_{G}$ beyond $v_{1} = V_{T}$. This ensures that the vector field is positive under the boundary control such that the target neuron reaches threshold while keeping the other neuron at $V_{G}$. Now if we assume that $\gamma_{1} \leq\frac{V_{T}}{V_{G}}$ (see Fig. 9(b), (c), (d)), then we can similarly show as in (88) that

89 $$ \dot{v}_{1}(u_{\mathrm{arc}})|_{v_{1} = V_{T}} < 0 $$

for this case, and we need to adopt the solution presented in Proposition 2 to fire Neuron 1 selectively. So we see that the nature of selective spiking solution, that is, (BANG/BANG-BOUNDARY) or (BANG/OFF-BANG), is contingent upon the ratio $\gamma_{1}$.

Figure 9 presents an intuitive representation of the geometric aspects of the solution space discussed in Sects. 3.1–3.3 with respect to $\gamma_{1}$, $\gamma_{2}$. Here, we analyze the pairwise feasibility for all possible parameter combinations. If $\gamma_{1} > \frac{V_{T}}{V _{G}}$ ($\Longrightarrow\gamma_{2} < \frac{V_{T}}{V_{G}}$) and Lemma 1 for Neuron 2 holds, then the neurons are pairwise feasible, that is, from any point in the phase plane we can fire either neuron selectively. Similarly, if we have $\gamma_{1} < \frac{V_{G}}{V _{T}}$ ($\Longrightarrow\gamma_{2} > \frac{V_{T}}{V_{G}}$), that is, Neuron 2 is Case 1, Neuron 1 is Case 2, and Lemma 1 holds for Neuron 1, then we can once again achieve pairwise feasibility. These two scenarios are depicted in Fig. 9(a), (d), respectively. When $\frac{V_{G}}{V_{T}}\leq\gamma_{1,2} \leq\frac{V_{T}}{V_{G}}$ (i.e., both neurons are Case 2), for pairwise feasibility, we must have Lemma 1 satisfy for each neuron individually. This creates a situation shown in Fig. 9(b), (c), where the separatrices for Neuron 1 and Neuron 2 intersect, which implies that, at the point of intersection, we have two different vector fields under the same control ($u = U$), which is a contradiction. Hence, if both neurons are Case 2, then we cannot have pairwise feasibility.

### A.3 Sketch of Regular Synthesis-Type Sufficient Conditions for Optimality

We outline regular synthesis-type sufficient conditions for optimality and how they apply to the optimal control problems considered in this paper. Any sufficiency theory in optimal control problems is based on the fact that the cost-to-go function ${\mathcal{V}}$ of the optimal control problem evaluated along extremal controls $u^{\ast}$ in a synthesis (to be verified as the optimal one), that is, the value of the objective is evaluated along the controls $u_{\ast}$ as a function of variable initial time $t_{0}$ and initial condition $\textbf{v}_{0}$, is, in a suitable way, a solution to the Hamilton–Jacobi–Bellman equation (38),

90 $$ \frac{\partial{\mathcal{V}}}{\partial t}(t, \textbf{v}) + \frac{ \partial{\mathcal{V}}}{\partial\textbf{v}} (t, \textbf{v}) f\bigl(t, \textbf{v}, u^{\ast}\bigr) + L\bigl(t, \textbf{v}, u^{\ast}\bigr) = 0. $$

If the cost-to-go function is continuously differentiable everywhere—and this is Bellman’s classical argument—then this is easy: given any other control *ũ* with corresponding trajectory $\tilde{\textbf{v}}$ defined over an interval $[t_{0},T]$, it follows that

91 $$\begin{aligned} \frac{\mathrm{d}}{\mathrm{d}t} {\mathcal{V}}\bigl(t,\tilde{ \textbf{v}}(t)\bigr) & = \frac{\partial {\mathcal{V}}}{\partial t}\bigl(t,\tilde{\textbf{v}}(t)\bigr) + \frac{\partial {\mathcal{V}}}{\partial\textbf{v}}\bigl(t,\tilde{\textbf{v}}(t) f\bigl(t, \tilde{\textbf{v}}(t) \bigr), \tilde{u}(t)\bigr) \\ & \geq-L\bigl(\tilde{\textbf{v}}(t), \tilde{u}(t)\bigr), \end{aligned}$$

and thus

92 $$ {\mathcal{V}}\bigl(T,\tilde{\textbf{v}}(T)\bigr)- { \mathcal{V}}(t_{0}, \tilde{\textbf{v}}_{0}) \geq- \int_{t_{0}}^{T}L\bigl(\tilde{\textbf{v}}(s), \tilde{u}(s)\bigr)\,\mathrm{d}s. $$

Hence

93 $$\begin{aligned} {\mathcal{V}} (t_{0},\tilde{ \textbf{v}}_{0}) & \leq \int_{t_{0}}^{T} L\bigl( \tilde{\textbf{v}}(s), \tilde{u}(s)\bigr)\,\mathrm{d}s + {\mathcal{V}} \bigl(T, \tilde{\textbf{v}}(T)\bigr) \\ & \leq \int_{t_{0}}^{T} L\bigl(\tilde{\textbf{v}}(s), \tilde{u}(s)\bigr)\,\mathrm{d}s + \varphi\bigl(T, \tilde{\textbf{v}}(T)\bigr) = J(\tilde{u}), \end{aligned}$$

and thus the cost $J(\tilde{u})$ along *ũ* is not better than the cost along the control $u^{\ast}$ given by ${\mathcal{V}}(t_{0}, \tilde{\textbf{v}}_{0})$. This proves the optimality of the controls in the synthesis.

The main issue with this argument is that problems for which the cost-to-go function is differentiable everywhere are generally rare. It is a much more common scenario that the value function loses differentiability on thin subsets, typically given by locally finite unions of embedded submanifolds of positive codimension. For the problems considered in this paper, such examples are given by the separatrix in Example 2 or a cut-locus that arises for the spike sequence $[2,1]$. In fact, such structures are omnipresent in solutions of time-optimal control problems.

This argument breaks down if there exist lower-dimensional submanifolds along which ${\mathcal{V}}$ is not differentiable since, in principle, the set of times when the controlled comparison trajectory $\tilde{\textbf{v}}$ lies in such a submanifold can be an arbitrary closed subset of the interval $[t_{0},T]$, and it is simply no longer possible to differentiate the function ${\mathcal{V}}$ along such a trajectory. Alleviating this issue is a highly nontrivial technical matter, which has led to *regular synthesis*-type arguments for optimality [39].

The key technical step is to perturb the given comparison trajectory $\tilde{\textbf{v}}$ in such a way that the perturbed trajectory has a cost that is close to $J(\tilde{u})$, whereas it meets the manifolds where ${\mathcal{V}}$ is not differentiable only in a finite set of times. It is clear that then the previous argument can be carried out piecewise, and the result follows in the limit as the approximations approach $\tilde{\textbf{v}}$. We briefly sketch the main steps of this reasoning, but refer the reader to Chap. 6.3 in [34], where the technical details of this argument are carried out in full.

In the first step, the comparison control *ũ* (a priori only a Lebesgue-measurable control) is approximated by a sequence $\tilde{u} _{n}$ of piecewise constant controls for which both the corresponding trajectories $\tilde{\textbf{v}}_{n}$ converge to $\tilde{\textbf{v}}$ uniformly over the interval $[t_{0},T]$ and the integrals converge as well,

94 $$ \int_{t_{0}}^{T}L\bigl(\tilde{\textbf{v}}_{n}(s), \tilde {u}_{n}(s)\bigr)\,\mathrm{d}s\rightarrow \int_{t_{0}}^{T}L\bigl(\tilde{\textbf{v}}(s), \tilde{u}(s)\bigr)\,\mathrm{d}s. $$

Note that the approximating controls are *piecewise constant*, that is, have only a finite number of switchings, and are not just simple measurable controls with a finite number of values as in the definition of Lebesgue measurability.

In the second step, the initial time $t_{0}$ and initial condition $\textbf{v}_{0}$ are perturbed so that the resulting trajectories $\tilde{\textbf{v}}_{n}$ only meet the submanifolds where the cost-to-go function ${\mathcal{V}}$ is not differentiable in at most a finite number of points. Essentially, this is just a transversality argument: since the perturbed controls are piecewise constant, trajectories are integral curves of smooth vector fields. If the flow of a vector field at a point *p* is transversal to an embedded submanifold *M* with positive codimension, then there exists $\varepsilon>0$ such that the flow does not lie in *M* for $0<\vert t\vert \leq \varepsilon $, and thus the corresponding time is an isolated point of the set of times when the flow meets *M*. We just need to make sure that there exist enough initial conditions for which this holds, and this can be guaranteed using Sard’s theorem [40].

As a consequence of all these perturbations, we need to take the limit as $n\rightarrow\infty$ and the trajectories generally no longer satisfy the terminal constraint. But all this works out in the limit if the cost-to-go function ${\mathcal{V}}$ is lower semicontinuous (and this is a necessary condition for it to be the value function of an optimal control problem in which the objective is minimized) and in addition satisfies the following two technical requirements:

1. For every constant admissible control *u*, the function ${\mathcal{V}}$ has the *no-downward-jumps* property along the vector field $x\mapsto f(x,u)$, that is, if *γ* is an integral curve of such a vector field defined on a compact interval $[a,b]$, $\gamma:[a,b]\rightarrow G$, $t\mapsto\gamma(t)$, then, for all $s\in(a,b]$, we have that
  95 $$ \lim_{h\searrow0}\inf \:\mathcal{V}\bigl(\gamma(s-h)\bigr) \leq{ \mathcal{V}}\bigl( \gamma(s)\bigr). $$
2. For every point *q* in the terminal manifold and every $\varepsilon >0$, there exists a nonempty open set $\varOmega\subset G\cap B_{\varepsilon }(q)$ such that, for all $z\in\varOmega$, we have that
  96 $$ \mathcal{V}(z)\leq\varphi(q)+\varepsilon. $$
  These two conditions are satisfied automatically wherever ${\mathcal{V}}$ is continuous.

For the problems considered in this paper, in Case 2 the cost-to-go function ${\mathcal{V}}$ need not be continuous, but it is not difficult to argue that condition (1) is satisfied. The value cannot decrease along constant controls as the separatrix would be crossed. This simply holds since trajectories can only cross from the region where the cost is smaller into the region where the cost is higher as the separatrix is defined by the maximum value of the control. Similarly, condition (2) is a weak continuity requirement that allows discontinuities in the value function at the terminal manifold. It only requires that, for any potential target point *q*, there exist sufficiently rich sets that are close such that the cost-to-go function ${\mathcal{V}}$ still has some upper continuity property along sequences converging to *q* in those sets. For our problems, this is satisfied by simply choosing *Ω* to lie in the regions where the optimal control is at its maximum value. Thus these conditions, albeit technical, are quite natural and generally are easily verified as is the case for our problems.

We close with some remarks about the synthesis in Case 1 when the trajectories contain the boundary arc. In this case, following the guard is in fact the *only* feasible control that the system can take, and thus, once the system hits the guard, the only control possible is the boundary control. This allows us to eliminate the state space constraint by extending the terminal set to include the guard $v_{2}=V_{G}$. We only need to define a penalty term $\varphi_{2}$ on the guard that represents the time it takes for the boundary control to reach the terminal state $v_{1}=V_{T}$. This transforms the time optimal control problem with state space constraint $v_{2} \leq V_{G}$ into an unconstrained optimal control problem whose terminal set consists of the union of two manifolds: the regular terminal manifold $v_{1}=V_{T}$ with penalty $\varphi_{1} \equiv0$ and the guard $v_{2}=V_{G}$ with penalty function $\varphi_{2}$. The two manifolds intersect at the point $[v_{1} \ v_{2}]^{T}=[V_{T} \ V_{G}]^{T}$, and $\varphi_{2}=0$ at this point. In this case, the cost-to-go function is not differentiable along the trajectory that ends in this point, but the conditions mentioned before are satisfied, and this is the optimal synthesis.

We emphasize that this argument is merely a convenient trick, which becomes available for this particular situation in Case 1. In general, however, that is, for Case 2 and the subsequent time optimal control problems of spike sequences, the methods and techniques sketched before are essential. Standard sufficiency arguments based on viscosity solutions to the Hamilton–Jacobi–Bellman equation fail once the cost-to-go function becomes discontinuous. This feature, related to questions of small-time local controllability (e.g., see [34, Sect. 7.2]), is no obstacle for synthesis type sufficiency arguments.

### A.4 Computation of *Λ* Controllable Sets

We now show the calculation for Neuron 1. There are two possible situations, namely, $\varLambda\leq T_{s}$ and $\varLambda> T_{s}$, which result in two different switching structures where $T_{s}$ denotes the time to reach $[V_{T}\ V_{G}]^{T}$ along the separatrix *Γ* from the initial condition

97 $$ \textbf{v}(0) = \bigl\{ (v_{1}, v_{2}): \textbf{v} \in \varGamma, v_{2} = 0\bigr\} . $$

If $\varLambda\leq T_{s}$, then we can find the neuron voltages $(v_{1}, v_{2})$ from which Neuron 1 reaches $V_{T}$, in time *Λ*,

98 $$ v_{1} = \mathrm{e}^{a_{1}\varLambda} \biggl(V_{T} - \frac{b_{1}}{a_{1}}U\bigl(1- \mathrm{e}^{-a_{1}\varLambda} \bigr)\biggr). $$

Note that $v_{2}$ does not come in (98) since for all $\textbf{v} \in\varGamma_{+}$, Neuron 1 reaches threshold without Neuron 2 hitting the guard.

For $\varLambda> T_{s}$, we assume that it takes *t̄* for Neuron 2 to hit the guard $V_{G}$ under bang control,

99 $$ \begin{aligned} V_{G} &= \mathrm{e}^{-a_{2}\bar{t}}v_{2} + \frac{b_{2}}{a_{2}}U\bigl(1- \mathrm{e}^{-a_{2}\bar{t}}\bigr), \\ \bar{t} &= \frac{1}{a_{2}}\log \biggl(\frac{v_{2} - \frac{b_{2}}{a_{2}}U}{V_{G}- \frac{b_{2}}{a_{2}}U} \biggr). \end{aligned} $$

The voltage of Neuron 1 at this time is calculated using (33). This means for $(v_{1}, v_{2})$ to be on the *Λ*-controllable set, Neuron 1 must reach the threshold $V_{T}$ in $(\varLambda- \bar{t})$ along the boundary arc, that is,

100 $$ \begin{aligned} V_{T} &= \mathrm{e}^{-a_{1}(\varLambda- \bar{t})}v_{1}(\bar{t}) + \frac{b _{1}}{a_{1}}U\bigl(1- \mathrm{e}^{-a_{1}(\varLambda- \bar{t})}\bigr). \end{aligned} $$

Simplifying (100), we get

101 $$ \begin{aligned} v_{1} = \mathrm{e}^{a_{1}\varLambda} \biggl( &V_{T} - \frac{b_{1}}{a_{1}}u _{\mathrm{arc}} - \mathrm{e}^{-a_{1}\varLambda}g(v_{2})\biggl(\frac{b _{1}}{a_{1}}U \biggl( 1- \frac{1}{g(v_{2})} \biggr) - \frac{b_{1}}{a_{1}}u _{\mathrm{arc}} \biggr) \biggr), \end{aligned} $$

where $g(v_{2}) = \bigl(\frac{v_{2} - \frac{b_{2}}{a_{2}}U}{V_{G}- \frac{b _{2}}{a_{2}}U} \bigr)^{\frac{a_{1}}{a_{2}}}$. From this we can find the *Λ* controllable set for the selective spiking of Neuron 1.

Similarly, for Neuron 2, we can find the set $\zeta_{2}(\varLambda)$.

### A.5 Calculation of off-Time for Fixed-Time Selective Spiking

In this section, we show how the off-time in (70) can be calculated to induce a spike in a specified time. Without loss of generality, we once again assume the target pattern $\varSigma_{P} = [(1, t_{1})]$, $\textbf{v}(0) = [v_{1}\ 0]^{T}$, and $t_{1} < T_{s}$. For the other cases, the computation is similar and follows from the optimal control structure discussed in Sects. 3.1 and 3.2. Let us denote the voltage at the end of the off segment $\textbf{v}(\hat{t}) = [\hat{v}_{1}\ 0]^{T}$. Now, using (42) in (70), we have

102 $$ \begin{aligned} &\frac{1}{a_{1}}\log \biggl( \frac{v_{1}}{\hat{v}_{1}} \biggr) - \frac{1}{a _{1}}\log\bigl(E(\hat{v}_{1}) \bigr) = t_{1}, \\ & \hat{v}_{1} = \frac{\frac{b _{1}}{a_{1}}U}{1 - (V_{T} - \frac{b_{1}}{a_{1}}U)\: \mathrm{exp}(a_{1}(t _{1} - \frac{1}{a_{1}}\mathrm{log}(v_{1})))}. \end{aligned} $$

Substituting $\hat{v}_{1}$ into $\hat{t}= \frac{1}{a_{1}}\log \big(\frac{v_{1}}{\hat{v}_{1}} \big)$, we get the desired off-time. Note that, for $t_{1} \gg T_{s}$, we will need to use the boundary segment in (102).

## width=LIF

LIF: Leaky Integrate-and-Fire
VP: Victor–Purpura
